# Therapeutic Management of Ocular Ischemia in Takayasu’s Arteritis: A Case-Based Systematic Review

**DOI:** 10.3389/fimmu.2021.791278

**Published:** 2022-01-14

**Authors:** Yue Zeng, Jianan Duan, Ge Ge, Meixia Zhang

**Affiliations:** ^1^ Department of Ophthalmology, West China Hospital, Sichuan University, Chengdu, China; ^2^ Research Laboratory of Macular Disease, West China Hospital, Sichuan University, Chengdu, China

**Keywords:** Takayasu’s arteritis, ocular ischemic syndrome, medical therapy, carotid surgery, endovascular procedures, systematic review

## Abstract

**Background:**

Takayasu’s arteritis (TA) is a rare, chronic granulomatous large-vessel vasculitis that can lead to ocular ischemia. Ocular outcomes after therapeutic management in TA remain largely unknown. We herein conduct a case-based systematic review to address the current treatment options in this particular cohort.

**Methods:**

PubMed, Medline, and EMBASE databases were searched pertaining to ocular outcomes after systemic treatment in TA. Studies reporting ocular examinations before and after treatment in TA patients with ocular ischemia were included. Clinical characteristics, therapies, ocular outcomes, and complications were recorded.

**Results:**

A 29-year-old woman with newly diagnosed TA showed dramatic regression of Takayasu’s retinopathy (TR) following balloon angioplasty. Optical coherence tomography angiography (OCTA) was used as a novel strategy for subsequent follow-up. A total of 117 eyes of 66 patients with a median age of 27 years were included for systematic review. TR was the most common ocular manifestation. Oral steroids were prescribed in nearly all patients (n = 65), followed by the use of methotrexate and antiplatelet therapy. Of the patients, 65.8% and 34.2% underwent open surgery and endovascular procedure, respectively. The median follow-up period was 12 weeks (interquartile range 8–33.5). Surgical therapy showed better ocular improvement (including visual and imaging responses) in both acute and chronic vision loss, along with fewer complications than medical therapy alone. In the surgical group, the visual prognosis was significantly better in patients with initial visual acuity better than 20/200 (p = 0.03) and those who underwent surgery before stage III TR (p = 0.01). Ocular outcomes were equivalent in the two surgical approaches.

**Conclusion:**

Clinicians should be familiar with ophthalmic manifestations of this potentially treatable complication in TA. Compared with medical therapy alone, surgical intervention might be a better choice for both acute and chronic vision loss. Surgery is best recommended before the onset of irreversible ischemia to the globe. A combined regimen (oral steroids, immunosuppressants, and antiplatelet drugs) might be effective for those with surgical contradictions or reluctance to an invasive procedure. Physicians should be aware of the importance of ocular examinations, including OCTA, during the diagnosis and follow-up in TA.

## Introduction

Takayasu’s arteritis (TA) is a rare, chronic granulomatous large-vessel vasculitis predominantly involving the aorta, its major branches, and pulmonary arteries. Although the etiology is poorly understood, it is thought that autoimmune cell-mediated response resulting in narrowing and obliteration of major arteries may be responsible for the disease (1). Systemic manifestations vary depending on the location of affected vessels and the severity of inflammation (2). Severe stenosis and occlusion of carotid arteries reduce blood supply to the eye and can lead to ocular ischemia, which has been described in 8.1%–68% of TA cases (3–5). The main retinal manifestation in response to ischemic insult is Takayasu’s retinopathy (TR) (6). Some other rare hypotensive complications are also described, such as anterior ischemic optic neuropathy (AION), retinal artery occlusion (RAO), and retinal vein occlusion (RVO) (7, 8). An early cohort study found that the 15-year survival was 66.3% versus 96.4% for TA patients with and without retinopathy (9). Mirouse et al. also reported that TR was independently associated with death and complication-free survival at 1 and 5 years (10). However, the systemic management of this particular complication in TA has long been overlooked. A comprehensive integration of current evidence is thus of urgent need.

Medical therapies, such as corticosteroids and conventional immunosuppressants (IS), have been the pillar of modern management of TA. However, approximately 20% of patients with vascular complications require surgical interventions (11). The recommended surgical approaches include the following: 1) open surgery, including bypass grafting surgery and endarterectomy; and 2) endovascular procedure, including percutaneous transluminal angioplasty with or without stent (12). Medications are always given as a perioperative adjunct in surgery-treated patients. It has been proposed that ocular hypoperfusion can be reversed following carotid reconstruction surgery (13, 14). However, surgical therapy in TA patients with ocular ischemia has rarely been documented. Compared with open surgery, angioplasty with or without stent is a less invasive and safe method, especially for short-segment lesions (15). Over the past decades, sporadic case reports have addressed ocular outcomes in surgery-treated TA patients (16–19). A recent case-series study (20) specifically reported the efficacy of endovascular stenting in TR patients. However, only one report focused on ocular outcomes following balloon angioplasty (8). Ocular prognosis varies between individual studies, in part due to different study populations and perioperative management. Although most previous studies have exclusively used fundus fluorescein angiography (FFA) for follow-up surveillance in TA, few studies looked at retinal perfusion in the macula, especially with the advent of optical coherence tomography (OCT) angiography (OCTA) (21, 22). A detailed summary of existing data is therefore necessary to clarify surgical outcomes in this particular cohort.

Herein, we describe an unusual case of TA with dramatic regression of hypotensive retinopathy following balloon angioplasty. OCTA was used as a novel strategy for subsequent follow-up. We also perform a comprehensive systematic review reporting the efficacy and safety of therapeutic management in TA patients with ocular ischemia, where surgical intervention is mainly focused.

## Case Presentation

A 29-year-old Chinese woman was referred to the ophthalmology clinic, complaining of gradual painless visual loss associated with intermittent episodes of whiteout of vision in both eyes for 1 month. Her visual disturbance was more prominent in the left eye and worsened upon standing. She also experienced headaches, dizziness, weakness in the left extremities, and jaw claudication while chewing. Two years prior to presentation, she had noted diminished left radial pulse. There was no contributory family history. She had been diagnosed with Type 5 TA 1 month prior to our ophthalmology clinic and received two courses of intravenous cyclophosphamide (CTX) with a 4-week interval followed by oral prednisone.

**Figure 1:**
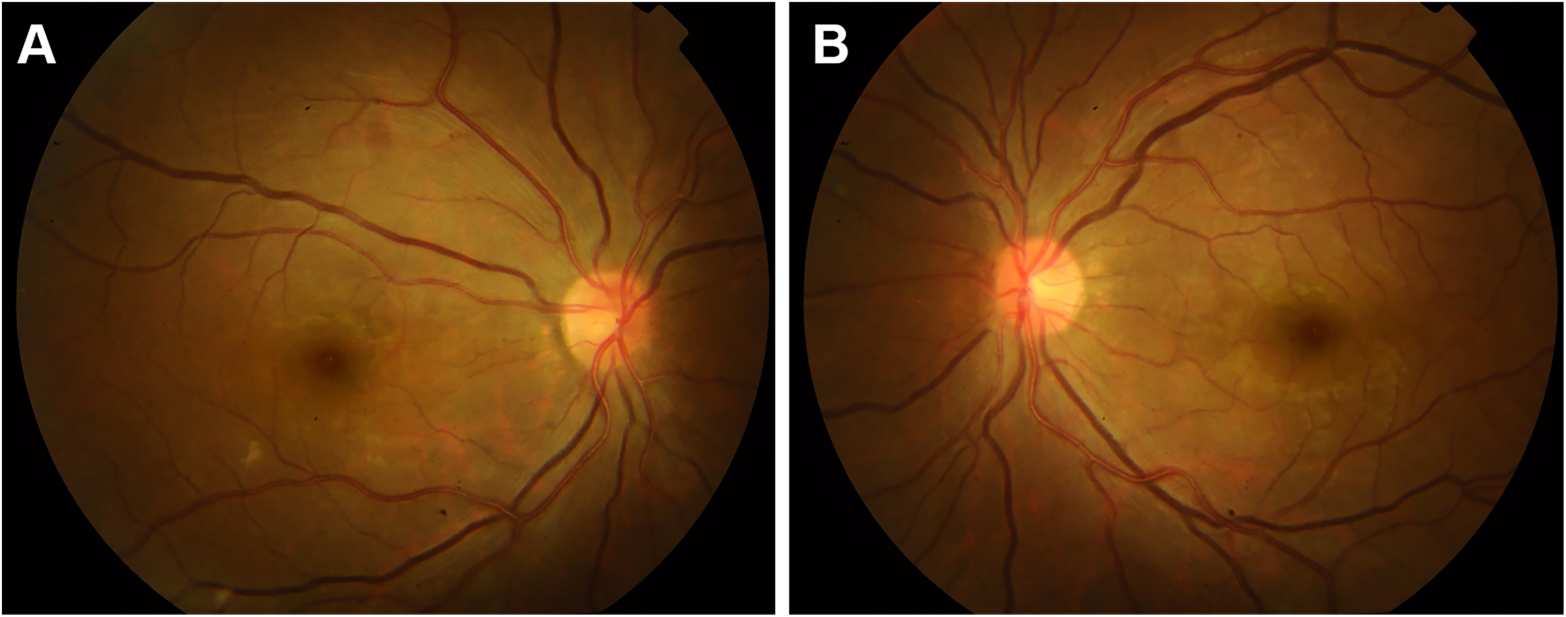
Retinal fundus photographs before surgery showing mild venous dilation in the right eye **(A)** and the left eye **(B)**.

At presentation, general physical examination disclosed the absence of left radial arterial pulses, along with feeble arterial pulses on her right side. Carotid and subclavian bruits were heard on both sides. Her blood pressure was 145/71 mmHg in the lower extremities. Ocular examination revealed a decimal best-corrected visual acuity (BCVA) of 0.02 and 0.4 in the right and left eyes. Intraocular pressure (IOP) was 6.3 mmHg in both eyes. Slit-lamp examination showed that anterior segment was unremarkable. Fundus examination revealed mildly distended veins in both eyes (**Figure 1**).

**Figure 2:**
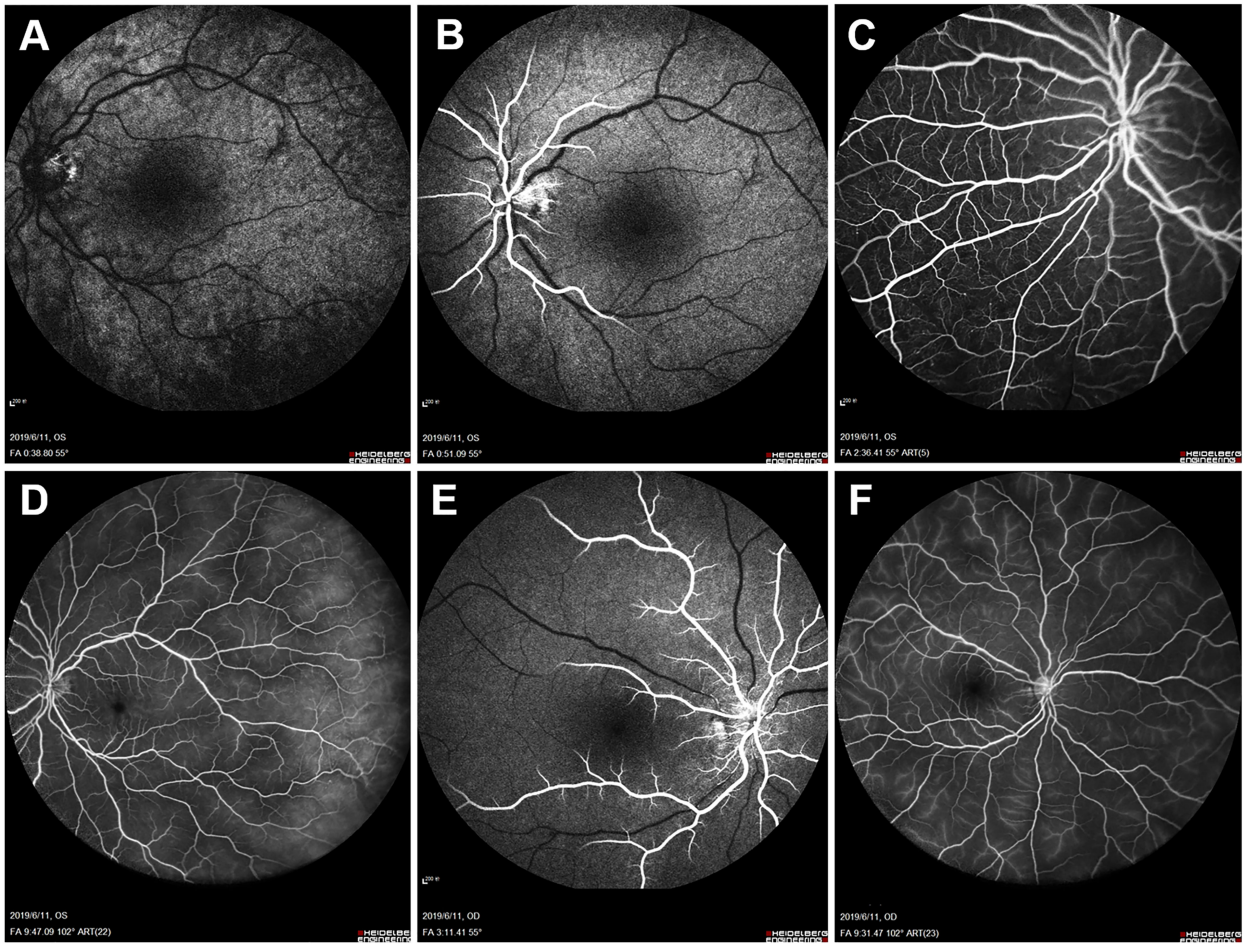
Baseline fundus fluorescein angiography (FFA). Left eye angiogram reveals **(A)** patchy choroidal filling and a prolonged arm-to-retina circulation time of 38 s. **(B)** Leading edge of fluorescein dye in the arteries. **(C)** Multiple microaneurysms in the midperipheral retina. **(D)** Diffuse dye leakage in the late phase with mildly dilated retinal veins. Right eye angiogram shows similar changes with **(E)** delayed arterial filling and **(F)** late-phase dye leakage.

**Figure 3:**
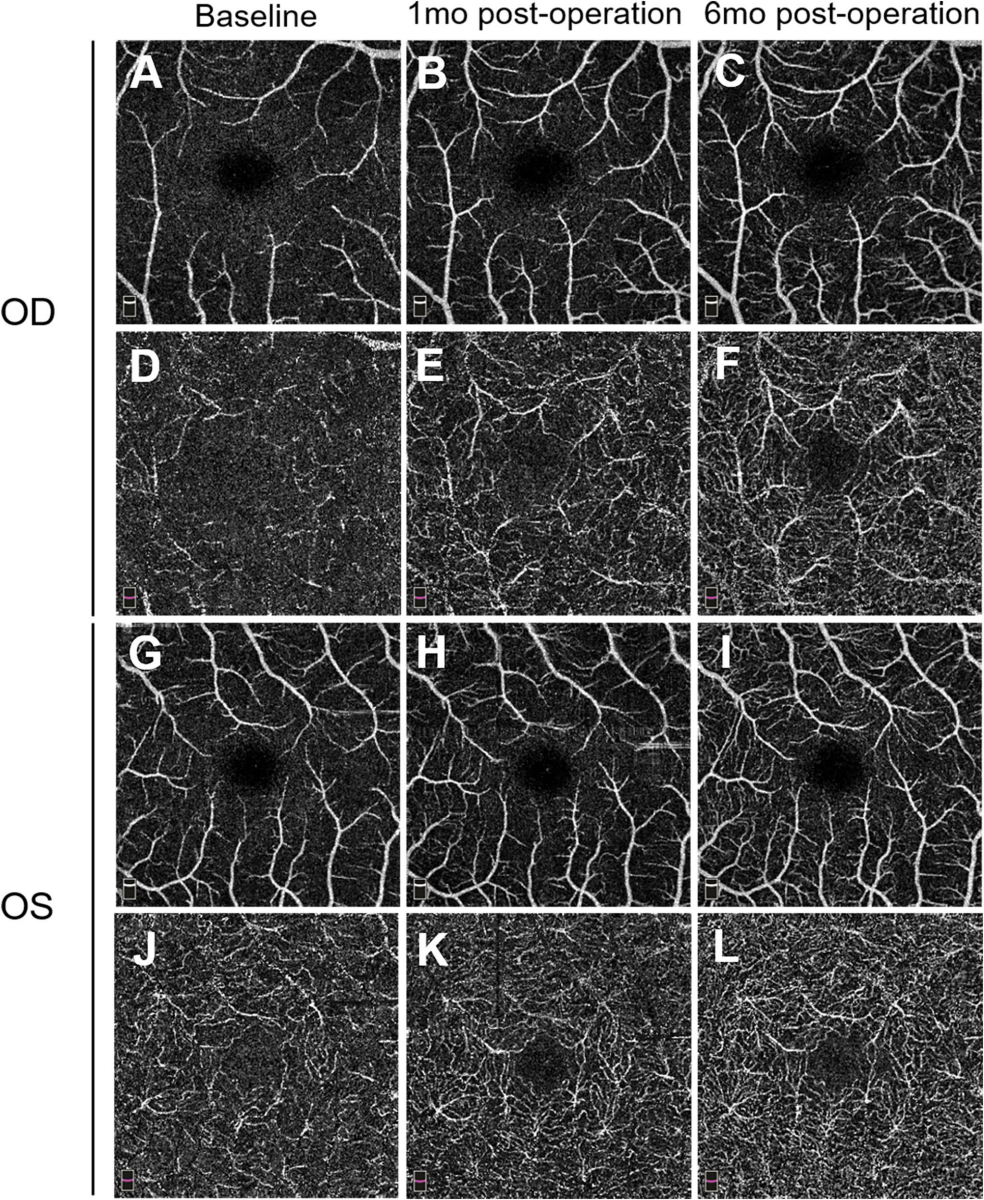
Pre- and postoperative 3.0 * 3.0 mm optical coherence tomography angiography (OCTA) images. Superficial capillary plexus (SCP) in the right eye **(A–C)** and left eye **(G–I)** preoperatively and at two follow-up visits (1 and 6 months) illustrate a gradual increase of macular vessel density. Deep capillary plexus (DCP) in the right eye **(D–F)** and left eye **(J–L)** reveals similar vascular restoration, along with reconstruction of the perifoveal anastomotic capillary arcade. mo, month(s).

FFA of the left eye showed delayed choroidal filling, leading edge of fluorescein dye in the arteries, prolonged arm-to-retina circulation time of 38 s, and arteriovenous transit time of 32 s. Diffuse microaneurysms were noted in the midperipheral area. In the late phase, retinal vessels were markedly stained. Similar changes were detected in the right eye. There was no evidence of capillary non-perfusion, arteriovenous shunts, or retinal neovascularization (**Figure 2**). Spectral domain OCT (SD-OCT) did not detect any significant macular changes. OCTA showed extreme rarefaction of perifoveal vascular density at the level of superficial capillary plexus (SCP) (**Figures 3A, G**) and deep capillary plexus (DCP) (**Figures 3D, J**) in both eyes. Disruption of the perifoveal vascular arcade was also noted predominately in DCP.

**Figure 4:**
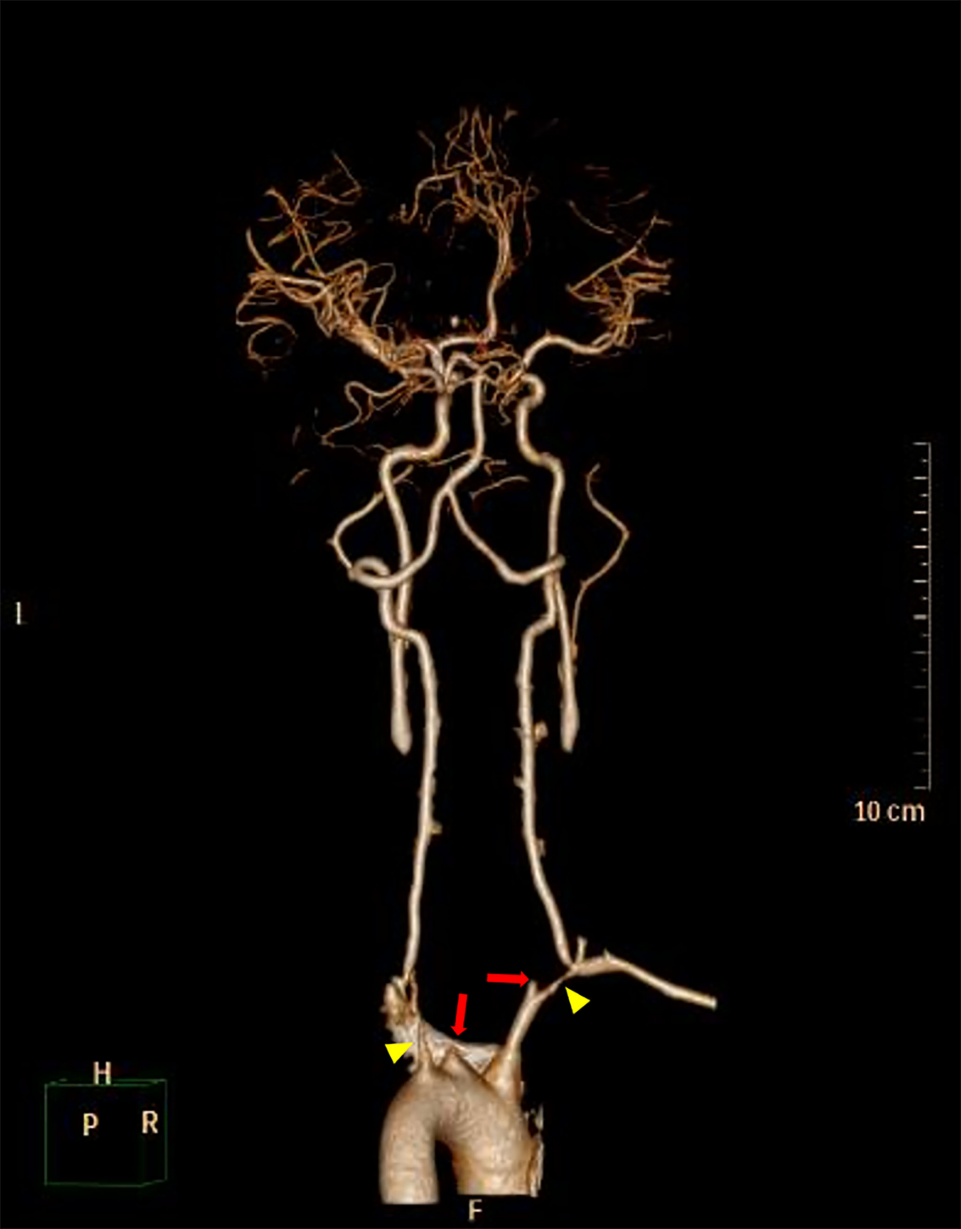
3D-reconstructed CT angiography (CTA) before balloon angioplasty shows complete occlusion of bilateral common carotid arteries (red arrows) and severe stenosis of bilateral proximal subclavian arteries (yellow arrowheads). Associated thrombi completely occluding bilateral common carotid arteries were also noted.

Blood testing on admission revealed normal erythrocyte sedimentation rate (8.0 mm/h), C-reactive protein (2.40 mg/L), and moderate eosinophilia (7,460 cells/μl). Chemical profile, liver and renal function, antinuclear antibody, antineutrophil cytoplasmic antibodies, and rheumatoid factor were normal. On vascular imaging, a carotid Doppler showed diffuse wall thickening with associated luminal stenosis involving bilateral common carotid arteries and subclavian arteries. High-grade stenosis of the bilateral proximal subclavian artery was noted. Marked luminal narrowing was seen in the right tibial artery in a peripheral ultrasound. A subsequent CT angiogram (CTA) disclosed circumferential thickening of thoracic aorta, complete occlusion of bilateral common carotid arteries, and critical stenosis of bilateral proximal subclavian arteries (**Figure 4**).

Based on the patient’s past history of TA, ocular symptoms, and relevant eye examinations, stage II TR was diagnosed according to the Umaya and Asayma classification (6). She was referred to a vascular surgeon and underwent balloon angioplasty of bilateral subclavian arteries. During the procedure, dilation of bilateral subclavian arteries was carried out with a 4 × 40 mm balloon through right femoral artery approach. Post-angioplasty angiogram showed markedly improved flow of pre-vertebral subclavian artery, predominately in the left side. She was put on prednisone (40 mg/day), CTX (50 mg/day), aspirin, and vasodilators afterward to induce remission in TA.

One month after surgery, the patient reported a slight improvement in her vision. Her blood pressure is 72/55 mmHg in the upper extremities. FFA revealed improved retinal blood flow, with a persistent number of microaneurysms and late-phase dye leakage in both eyes. Paralleled to FFA findings, OCTA showed considerably increased vascular density in the SCP (**Figures 3B, H**) and DCP layer (**Figures 3E, K**). She remained on tapered prednisone (35 mg/day), CTX (50 mg/day), vasodilator (40 μg twice daily), and aspirin.

**Figure 5:**
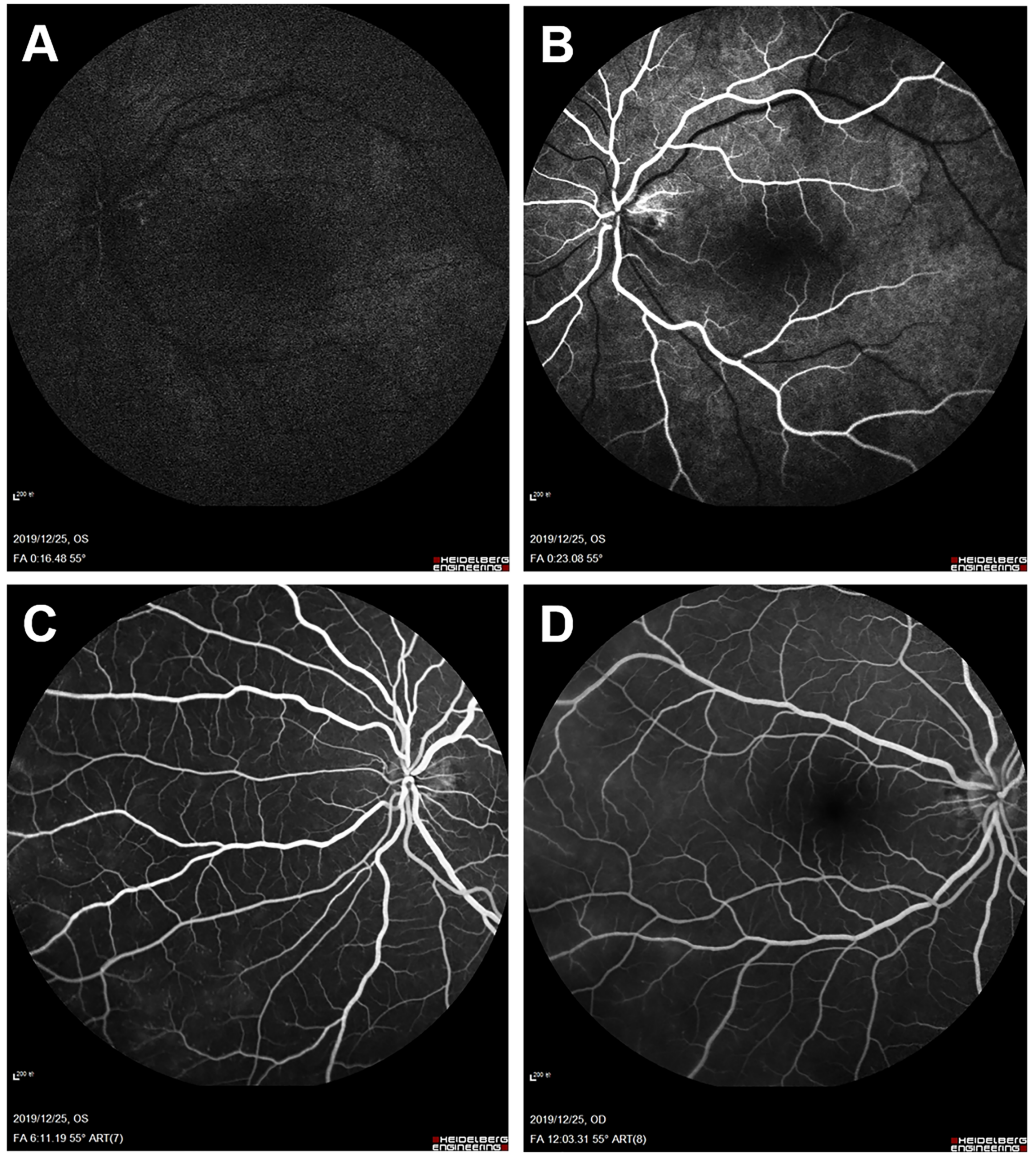
Fundus fluorescein angiography (FFA) 6 months after balloon angioplasty. Left eye angiogram reveals **(A, B)** restoration of retinal blood flow (decreased arm-to-retina circulation time to 16 s) and **(C)** decrease of microaneurysms with less evident leakage in the late phase. **(D)** Right eye angiogram in the late phase shows similar improvement.

On follow-up of 6 months, she continued to sustain good improvement in symptoms. Her BCVA improved to 0.6 in the right eye and remained stable at 0.4 in the left eye. IOP was 9 mmHg in both eyes. A fundus examination revealed a superficial hemorrhage in the peripheral retina of the left eye. Repeated FFA of the left eye showed a decrease of arm-to-retina circulation time and arteriovenous transit time to 16 and 8 s, respectively. The number of microaneurysms decreased, with less evident dye leakage in the midperipheral area. Right eye angiogram showed similar improvement (**Figure 5**). No capillary non-perfusion and retinal neovascularization were observed. Notably, OCTA revealed further normalization of vascular density, along with restoration of perifoveal anastomotic capillary arcade (**Figures 3C, F, I, L**). CTA revealed moderate restoration of flow in the bilateral common carotid arteries and subclavian arteries.

## Materials and Methods

### Search Strategy

To identify publications analyzing ocular outcomes in TA patients, a literature search was performed based on the Preferred Reporting Items for Systematic Reviews and Meta-Analyses (PRISMA) statement (www.prisma-statement.org). We conducted a systematic search in PubMed, Medline, and EMBASE databases from inception to August 2021. The following keywords including “eye disease,” “ocular,” or “retinopathy” and “Takayasu’s arteritis” or “Pulseless disease” were used in PubMed search. The search strategy for Medline and EMBASE database was adapted from the initial PubMed strategy. Manual search from references of extracted papers was performed to identify relevant publications. Only studies published in English and Chinese were accepted.

### Selection Criteria

Articles were considered for inclusion if they were case reports, case series, observational studies, or randomized controlled trials (RCTs). Letters or correspondences addressing relevant cases were also included. Review articles, conference abstracts, commentaries, and animal studies were excluded. Studies that 1) reported TA patients presenting with ocular ischemic symptoms, 2) reported a clearly defined treatment protocol for each patient, including either medical therapy or surgical interventions, and 3) reported patients with ocular outcome data, including visual acuity, fundus examinations, FFA, color Doppler imaging (CDI), OCT, or OCTA, were considered to satisfy the inclusion criteria. Since the review focused mainly on ocular ischemia in TA patients, studies were excluded if study subjects were diagnosed with hypertensive retinopathy or other non-ischemic eye diseases (i.e., uveitis, scleritis, and glaucomatous optic neuropathy). Studies that reported anecdotal data on ocular symptoms, instead of the aforementioned ocular examinations, were also excluded to reduce bias.

### Data Extraction and Quality Assessment

Two reviewers (YZ and JD) independently screened titles and abstracts of potentially relevant articles. If necessary, full texts were extracted and checked for eligibility criteria. Discrepancies were resolved through consensus and consulting a third reviewer (GG). TA patients with ocular ischemia were selected from eligible articles. The following information was extracted: author, year of publication, study origin, subject age, subject sex, initial visual acuity, preoperative ocular examinations, collateral vessels formation, diagnosis, surgical procedure, medical therapy, ocular procedure, follow-up duration, postoperative ocular outcomes, and complications. TA was characterized based on the classification of Hata et al. (23), as follows: Type 1, branches of aortic arch; Type 2a, ascending aorta and/or its branches; Type 2b, descending thoracic aorta, ascending aorta, and/or its branches; Type 3, descending thoracic aorta, abdominal aorta, and/or renal arteries; Type 4, abdominal aorta, and/or renal arteries; and Type 5, combined feature of Type 2b and 4. We used the Umaya and Asayma classification (6) to standardize the severity of TR. Clinical response was defined as an improvement of visual acuity or ocular symptoms after treatment. Imaging response was defined as resolution of retinopathy on FFA (a gold standard for retinopathy diagnosis). Ocular improvement was defined as either clinical or imaging response achieved.

Methodological quality was assessed using the Newcastle–Ottawa scale (NOS) for case series and case reports adapted by Murad et al. (24). The tool consists of 8 criteria out of 4 domains (selection, ascertainment, causality, and reporting). Items that related to adverse drug events were removed and resulted in 5 criteria to be assessed. We considered the study as “good quality” when all five criteria were satisfied, “moderate quality” when four were satisfied, and “poor quality” when three or fewer were satisfied.

### Statistical Analysis

Statistical analysis was performed using SPSS v.25.0 (SPSS Company, Chicago, IL, USA). Categorical variables were presented as numbers and percentages. Continuous variables were presented as the median and interquartile range (IQR). Normality of data distribution was assessed by the Shapiro–Wilk test. Categorical variables between the groups were compared with the chi-squared test or Fisher’s exact test. Continuous variables were compared with the Wilcoxon rank-sum test. A p-value was only calculated in analyses with a minimum of 11 observations per group for reliable comparisons, where p < 0.05 was considered statistically significant.

## Results

### Literature Search Results

**Figure 6:**
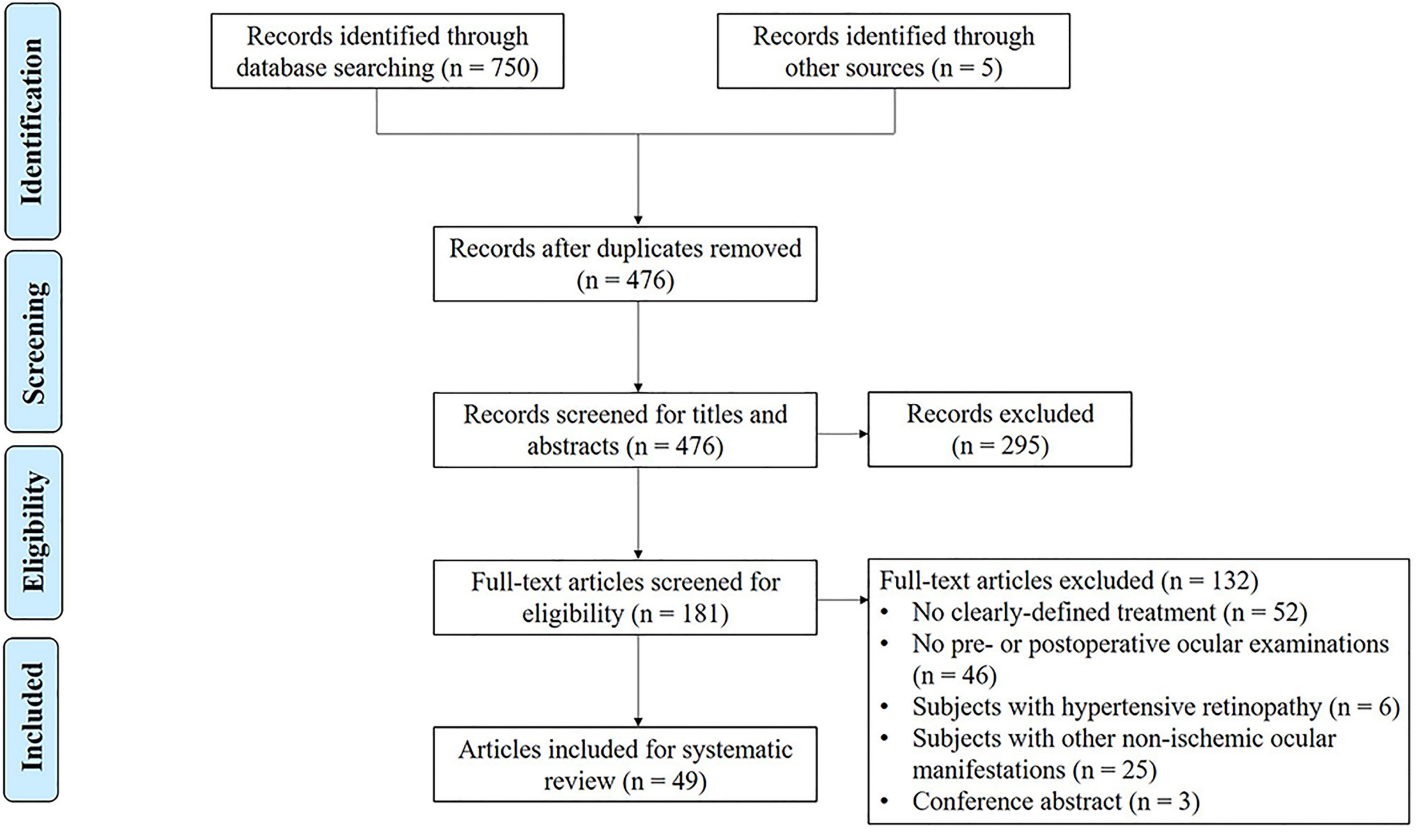
Preferred Reporting Items for Systematic Reviews and Meta-Analyses (PRISMA) flowchart of the study selection process.

As shown in **Figure 6**, the strategy yielded 750 articles (200 articles from PubMed, 268 articles from Medline, and 282 articles from EMBASE). Five additional studies were identified from a manual search of references. After duplicates were removed, 476 articles were retrieved and screened for titles and abstracts. Afterward, full texts from 181 citations were obtained, and 114 additional articles were excluded for the following reasons: subjects without clearly defined treatment protocol (52 articles), subjects without ocular examinations (46 articles), subjects with hypertensive retinopathy (6 articles), subjects with other non-ischemic ocular manifestations (25 articles), and conference abstracts (3 articles). Consequently, 115 study eyes (65 patients) from 49 publications were included in the analysis. Among them, 2 were written in Chinese (6 patients) (18, 25), and the remaining 47 (59 patients) were published in English. Included studies were published between 1957 and 2021, the types of which were case reports (40 articles) (7, 8, 16, 17, 19, 25–58), case series (7 articles) (3, 17, 18, 20, 59–61), and image-based articles (2 articles) (21, 62). The methodological quality assessment showed that 8 studies were of good quality, 33 studies were of moderate quality, and 8 studies were of poor quality (**Supplementary Table 1**).

### Demographic Characteristics and Clinical Features

Characteristics of studies are summarized in **Supplementary Tables 2**, **3**. Data from our case were added to the identified 65 patients for global analysis, and the clinical characteristics of overall 66 cases (117 eyes) are summarized in **Table 1**. The analysis showed that 61 patients (92.4%) were women with a median age at ocular diagnosis of 27 years (IQR: 20.8–35). A total of 54 patients (81.2%) were of Asian ethnicity. The most common systemic symptoms were cerebrovascular signs found in 35 cases (67.3%). Progressive visual loss presented in 29 cases (48.3%), followed by amaurosis fugax in 21 (35%), sudden vision loss in 17 (28.3%), and floaters in 3 (5%). Visual symptoms were the first presenting manifestations in 4 cases (6.6%). All of the reported cases had aortic arch branches involvement, and TA was extended (type V) in 11 cases. Collateral vessels to the occluded carotid artery were observed in 9 out of 58 cases (15.5%).

**Table 1:** Summary of clinical characteristics of 66 cases (117 eyes).

Variable	Total	Surgical therapy	Medical therapy	p-Value^a^
Total number of patients	66	37	29	
Age (years)	27 [20.8–35]	28 [24.5–34]	24 [18–37]	0.13
Gender				
Male	5 (7.6)	3 (8.1)	2 (6.9)	1.00
Female	61 (92.4)	34 (91.9)	27 (93.1)	
Ethnicity				
Asian	54 (81.2)	31 (83.8)	23 (79.3)	0.76
Non-Asian	12 (18.8)	6 (16.2)	6 (20.7)	
Systemic symptoms	52	25	27	
Constitutional symptoms	22 (42.3)	3 (11.5)	19 (65.5)	<0.001^*^
Cerebrovascular symptoms	35 (67.3)	20 (76.9)	15 (51.7)	0.08
Cardiovascular symptoms	1 (1.9)	1 (3.8)	0	0.49
Limb claudication	15 (28.8)	8 (30.8)	7 (24.1)	0.77
Pulselessness	26 (50)	11 (42.3)	15 (51.7)	0.41
Visual symptoms	60	31	29	
Progressive visual loss	29 (48.3)	17 (54.8)	12 (41.4)	0.32
Amaurosis fugax	21 (31.8)	12 (38.7)	9 (31)	0.60
Sudden visual loss	17 (25.8)	4 (12.9)	12 (41.4)	0.02^*^
Floaters	3 (4.5)	2 (6.5)	1 (3.4)	1.00
Type of TA	59	30	29	
Type I	42 (71.2)	24 (80)	28 (71.8)	0.13
Type II	6 (10.2)	4 (13.3)	2 (5.1)	
Type III	0	0	0	
Type IV	0	0	0	
Type V	11 (18.4)	2 (6.7)	9 (23.1)	
Collateral formation	58	29	29	
Yes	9 (15.5)	6 (20.7)	3 (10.3)	1.00
No	49 (84.5)	23 (79.3)	26 (89.7)	
Medical therapy	35	16	29	
Oral corticosteroids	34 (97.1)	15 (93.8)	29 (100)	0.36
Intravenous pulse with MP	11 (31.4)	2 (12.5)	9 (31)	0.28
Immunosuppressive agents				
Methotrexate	14 (40)	4 (25)	10 (34.5)	0.74
Azathioprine	4 (11.4)	1 (6.3)	3 (10.3)	1.00
Oral cyclophosphamide	6 (17.1)	4 (25)	2 (6.9)	0.17
Pulse cyclophosphamide	2 (5.7)	2 (12.5)	0	0.12
Mycophenolate mofetil	3 (8.6)	1 (6.3)	2 (6.9)	1.00
Infliximab	2 (5.7)	1 (6.3)	1 (3.4)	1.00
Antiplatelet therapy	11 (31.4)	4 (25)	7 (24.1)	1.00
Anticoagulants	3 (8.6)	1 (6.3)	2 (6.9)	1.00
Surgical therapy				
Open surgery	25 (37.9)	25 (67.6)	–	–
Endovascular surgery	12 (18.2)	12 (32.4)	–	
Follow-up period (weeks)^b^	12 [8–33.5]	20 [8–48]	12 [8–23]	0.48
Total number of eyes	117	66	51	
TR stage	99	60	39	
Stage I	2 (2)	2 (3.3)	0	0.61
Stage II	40 (40.4)	26 (43.3)	14 (35.9)	
Stage III	15 (15.2)	10 (16.7)	5 (12.8)	
Stage IV	37 (37.4)	19 (31.7)	18 (46.2)	
NA	5 (4.3)	3 (5)	2 (5.1)	
Local therapy				
Photocoagulation	24 (20.5)	11 (16.7)	13 (25.5)	0.26
Intravitreal anti-VEGF	7 (6)	2 (3)	5 (9.8)	0.24
Vitrectomy	3 (2.6)	0	3 (5.9)	0.08
Cataract surgery	5 (4.3)	2 (3)	3 (5.9)	0.65
Anti-glaucoma therapy	2 (1.7)	2 (3)	0	0.50
Small vessel involvement				
AION	8 (6.8)	3 (4.5)	5 (9.8)	
RAO	9 (7.7)	3 (4.5)	6 (11.8)	
RVO	1 (0.9)	0	1 (2)	

Data are presented as median [interquartile range] or number (percentage).

TA, Takayasu’s arteritis; TR, Takayasu’s retinopathy; MP, methylprednisolone; AION, anterior ischemic optic neuropathy; VEGF, vascular endothelial growth factor; RAO, retinal artery occlusion; RVO, retinal vein occlusion; F, female; M, male.

a Statistical analysis was made between surgical therapy and medical therapy groups. Only variables with a minimum of 11 observations per group were analyzed.

b Follow-up period was documented in 51 patients.

^*^p < 0.05, which indicated statistical differences between groups.

Including our case, 52 patients (99 eyes, 78.8%) were assumed to have TR, among which 2 eyes (2%) were at stage I, 40 eyes (40.4%) were at stage II, 15 eyes (14.1%) were at stage III, and 37 eyes (38.4%) were at stage IV. Of note, two cases with bilateral TR had associated AION (21, 50). Stage of retinopathy was not identified in 3 cases due to delayed presentation (27) or lack of detailed fluorescein angiogram (48, 59). The remaining 18 eyes of 14 patients were diagnosed with RAO (9 eyes, 7.6%), AION (8 eyes, 6.7%), or RVO (1 eye, 0.8%). TA duration before treatment ranged from 0 to 30 years, and TA was inaugural in 39 cases. No significant differences in demographic features (age, sex, and ethnicity distribution), retinal manifestations, and follow-up period were found between patients treated with medical therapy alone and those with surgery.

### Therapy

There were 25 articles, consisting of 29 patients (51 eyes), that explored the efficacy of medical therapies alone on ocular ischemia. Almost all patients (28/29, 96.6%) were treated with oral corticosteroids. Of them, intravenous pulse methylprednisolone (MP) was previously administrated to 9 patients (31%). One study published in 1957 reported a case treated solely with anticoagulation drugs and vasodilators (58). Additional IS were prescribed to 16 patients (55.2%), wherein oral methotrexate was the most commonly used (10/29, 34.5%). Alternative IS were included: azathioprine in 3 patients (10.3%), oral CTX in 2 patients (6.8%), mycophenolate mofetil in 2 patients (6.8%), and leflunomide in 1 patient (3.4%). Infliximab was added in one patient with RAO due to sudden bilateral blindness (3.4%). Antiplatelet and anticoagulation agents were used in 7 (24.1%) and 2 patients (6.8%), respectively. Adjunctive local therapy was administered in 19 eyes of 11 patients (37.9%), wherein photocoagulation in 13 eyes, anti-vascular endothelial growth factor (anti-VEGF) therapy in 5 eyes, vitrectomy in 3 eyes, and cataract surgery in 3 eyes. The median follow-up period was 12 weeks (IQR: 8–23).

The remaining 24 articles focused on surgical management of ocular ischemia in TA. Surgery was performed in 37 patients (66 eyes) with a median follow-up period of 20 weeks (IQR: 8–48). Among them, 25 patients (67.6%) and 12 patients (32.4%) underwent open surgery (bypass grafting in 24, endarterectomy in 1) and endovascular surgery (stenting in 10, balloon angioplasty in 2), respectively. Aorto-carotid bypass grafting was the most common surgery performed. Adjunctive medical therapy was documented in 16 patients. Oral corticosteroids were prescribed in all patients, 2 of which received intravenous pulse MP at a dose of 1,000 mg preoperatively. IS consisted of methotrexate in 4 (25%), oral CTX in 4 (25%), intravenous pulse CTX in 2 (12.5%), azathioprine in 1 (6.3%), and mycophenolate mofetil in 1 (6.3%). Infliximab was added in one patient with TR (27). Photocoagulation was performed perioperatively in 11 eyes of 6 patients (16.7%) with TR of stage III and above. One patient received intravitreal anti-VEGF bilaterally to treat neovascular glaucoma (NVG). Of note, local therapies were less performed compared with the medical therapy group.

### Outcomes of Medical Therapy Alone

We next evaluated responses in TR patients treated with medical therapy alone. Of note, since a different pathogenesis might be expected in TA patients manifesting with acute retinal ischemia or small vessel occlusion (i.e., AION, RAO, and RVO) (61), these patients were omitted from the main cohort for separate descriptions. During follow-up, clinical response was achieved in 13 eyes (43.3%) of 8 patients. Vision was stabilized in 13 eyes (43.3%) and worsened in 4 eyes (13.3%). Imaging response was investigated in 15 eyes of 9 patients. Regression of retinopathy was achieved in 9 eyes (60%), wherein 1/4 for stage II, 2/2 for stage III, and 6/9 for stage IV. It is noteworthy that poor compliance to medication might contribute to the subsequent non-response in one case with bilateral stage II TR (38). Among responders, regressed new vessels were the most common findings observed in 5 eyes (55.6%), in part due to the administration of intravitreal anti-VEGF and photocoagulation.

Oral steroids were prescribed to all patients diagnosed with acute retinal ischemia. In four cases with AION, 2 were additionally administered with intravenous pulse MP, and 1 was treated with methotrexate and antiplatelet agents (50, 56, 57). Although visual improvement was achieved in one patient on oral steroids alone (17), none of them showed imaging responses. RAO was diagnosed in 5 patients. Improved visual acuity was reported in 2 patients treated with high-dose oral steroids, while one of them developed NVG (45, 54). Noel et al. (61) reported a case showing ocular aggravation in 2 months despite the combination of pulse MP and methotrexate, which stabilized after infliximab was added. The remaining 2 patients (one with the combination of pulse MP and methotrexate) became completely blind after treatments (46, 55). The only patient diagnosed with RVO was treated with oral steroids and methotrexate, showing stabilized vision despite the occurrence of steroid-related glaucoma and cataract (61).

We further compared clinical data between TR patients with and without ocular improvement (as defined in the *Materials and Methods* section) (**Table 2**). Overall, ocular improvement was observed in 18 eyes of 10 patients (50%). The median duration of medical treatment was similar between groups. Improved patients have been less frequently treated with methotrexate (10% of patients versus 60% of non-improved patients), while aspirin was used more frequently (50% of patients versus 20% of non-improved patients). Eye-level analysis revealed that initial visual acuity was better in improved eyes with a median logMAR 0.5 (IQR: 0–3.2) versus logMAR 1.8 (IQR: 0.7–3.1) in the non-improved eye, although no significant difference was observed (p = 0.27). Other factors, including TR stage and local therapy, were not significantly associated with ocular outcomes in medicated patients.

**Table 2:** Comparison of TR patients with and without ocular improvement after medical therapy alone.

Variables	Improved	Not improved	p-Value^a^
Number of patients	10	10	–
Age (years)	23 [17–26]	22 [18–32]	
Males	1 (10)	1 (10)	
Duration of medication (weeks)^b^	12 [4–24]	12 [8–27]	
Systemic symptoms	9	9	
Constitutional	7 (77.8)	7 (77.8)	
Vascular	7 (77.8)	8 (88.9)	
Type of TA			
Type I	7 (70)	5 (50)	
Type II	0	1 (10)	
Type V	3 (30)	4 (40)	
Oral steroids alone	2 (20)	2 (20)	
Intravenous pulse with MP	2 (20)	2 (20)	
Non-steroid immunosuppressants			
Any	7 (70)	6 (60)	
Methotrexate	1 (10)	6 (60)	
Azathioprine	2 (20)	1 (10)	
Oral cyclophosphamide	2 (20)	0	
Intravenous pulse cyclophosphamide	0	1 (10)	
Mycophenolate mofetil	1 (10)	1 (10)	
Antiplatelet agents	5 (50)	3 (30)	
Anticoagulants	0	2 (20)	
Collateral formation	1 (10)	1 (10)	
Number of eyes	18	21	
Initial visual acuity (logMAR)	0.5 [0–3.2]	1.8 [0.7–3.1]	0.27
≥20/200	11 (61.1)	8 (38.1)	0.21
<20/200	7 (38.9)	13 (61.9)	
TR stage	18	19	
<Stage III	6 (33.3)	8 (42.1)	0.74
≥Stage III	12 (66.7)	11 (57.9)	
Local therapy			
Any	8 (44.4)	7 (33.3)	0.53
Photocoagulation	3 (16.7)	6 (28.6)	0.46
Intravitreal anti-VEGF	3 (16.7)	1 (4.7)	0.32
Vitrectomy	3 (16.7)	0	0.09
Cataract surgery	2 (11.1)	1 (4.8)	0.59

Data are presented as median [interquartile range] or number (percentage).

TA, Takayasu’s arteritis; MP, methylprednisolone; TR, Takayasu’s retinopathy; VEGF, vascular endothelial growth factor.

a Statistical analysis was only made in eye-level comparison, as the sample size in patient-level analysis (n = 10 per group) was too small for reliable statistical comparisons.

b Duration of medication was available in 18 patients.

### Outcomes of Surgical Therapy

During follow-up, clinical response was investigated in 52 eyes of 28 patients. Among them, 30 eyes (57.7%) experienced visual improvement, 13 eyes (25%) showed stable vision, and only 9 eyes (17.3%) worsened. There was no patient complaint of amaurosis fugax during the follow-up period. Interestingly, 2 eyes with TR showed improved visual function even after operation at the opposite side of the involved eye (19, 32). Imaging response was documented in 41 eyes of 23 TR patients. Regression of retinopathy was noted in 36 eyes (87.8%), wherein 1/1 for stage I, 19/19 for stage II, 5/7 for stage III, and 10/13 for stage IV. Of particular interest, restoration of retinal perfusion (21 eyes, 53.8%) was the most common findings associated with angiogram improvement, followed by resolution of microaneurysms (20 eyes, 48.8%), regressed new vessels (4 eyes, 9.8%), decreased capillary non-perfusion (3 eyes, 7.3%), less dilated retinal veins (2 eyes, 4.9%), and resolved arteriovenous anastomosis (1 eye, 2.4%). Four cases had CDI performed pre- and postoperatively, showing marked improvement of hemodynamics in the ophthalmic artery (27, 32, 34, 34). One case with bilateral stage IV TR showed complete regression of optic disc neovascularization on OCTA after carotid endarterectomy (21). Of note, two eyes contralateral to the surgical side had unexpected visual improvement.

In 5 eyes (3 patients) that presented with AION, 4 eyes experienced visual improvement (8, 21), while 1 eye remained stable (60). Consequently, all eyes showed optic disc pallor after surgery. RAO was diagnosed in 3 patients unilaterally, all of which experienced good improvement in vision (25, 26, 59).

We further compared clinical data between TR patients with and without ocular improvement (**Table 3**). Overall, ocular improvement was noted in 25 patients (78.1%). The median follow-up period in improved patients [20 weeks (IQR: 8–28)] was slightly longer than that in non-improved patients [14 weeks (IQR: 1–65)]. It is worth noting that patients who underwent open and endovascular surgery had similar improvements (70.8% versus 71.4%). Adjunctive medical therapy was documented in 14 patients, revealing that improved patients were less frequently treated with methotrexate (11.1% of patients versus 60% in non-improved patients). Surprisingly, three patients administered with oral steroids alone postoperatively showed amelioration of stage III and IV TR. Koz et al. reported one case who switched from methotrexate to pulse CTX because of a cerebrovascular accident and eventually died from bypass surgery (31). Other medical therapies were similar between groups. The visual prognosis was better in patients with collateralization (27.8% versus 16.7%). Other factors such as age, sex, type of TA, and systemic symptoms were not significantly associated with ocular outcomes. Eye-level analysis was further performed in 62 study eyes, 37 of which (59.7%) showed amelioration of ocular ischemia postoperatively. Initial median visual acuity was better in improved eyes, with a median of 0.5 logMAR (IQR: 0–1) compared with 1.0 logMAR (IQR: 0–2.9) in non-improved eyes, although no significant difference was found. Eyes with initial visual acuity better than 20/200 Snellen equivalent had significantly better visual outcomes (p = 0.03). Improved TR eyes were significantly less severe (stage I or II) before the procedure than non-improved eyes (p = 0.01). Local therapy, commonly used for patients with severe ocular ischemia, was beneficial for regression of retinal neovascularization as noted in 2 cases (19, 21). However, 2 cases went on to develop NVG despite the introduction of photocoagulation and intravitreal anti-VEGF therapy (28, 34). Although Dogra et al. (20) reported that eyes undergoing endovascular surgery after a gap of 3 months might have worse outcomes than earlier ones, it was not summarized in our study, as staged procedures were not indicated in most studies.

**Table 3:** Comparison of TR patients with and without ocular improvement after surgical therapy.

Variables	Improved	Not improved	p-Value^a^
Number of patients	25	7	–
Age (years)	28 [25–36]	29 [25–33]	
Males	1 (4)	1 (14.3)	
Follow-up period (weeks)	20 [8–28]	13.5 [1.1–64.5]	
Systemic symptoms	19	6	
Constitutional	2 (10.5)	1 (16.7)	
Vascular	14 (73.7)	6 (100)	
Type of TA	19	6	
Type I	14 (73.7)	6 (100)	
Type II	3 (15.8)	0	
Type V	2 (10.5)	0	
Surgical procedure	24	7	
Open surgery	17 (70.8)	5 (71.4)	
Endovascular surgery	7 (29.2)	2 (28.6)	
Adjunctive medical therapy	9	5	
Oral steroids alone	3 (33.3)	0	
Intravenous pulse with MP	2 (22.2)	0	
Non-steroid immunosuppressants			
Any	5 (55.6)	4 (80)	
Methotrexate	1 (11.1)	3 (60)	
Azathioprine	1 (11.1)	0	
Oral cyclophosphamide	2 (22.2)	2 (40)	
Intravenous pulse cyclophosphamide	1 (11.1)	1 (20)	
Mycophenolate mofetil	1 (11.1)	0	
Infliximab	1 (11.1)	0	
Antiplatelet agents	6 (66.6)	3 (60)	
Collateral formation ^b^	5 (27.8)	1 (16.7)	
Number of eyes	37	25	
Initial visual acuity (logMAR)^c^	0.5 [0–1]	1.0 [0–2.9]	0.17
≥20/200	29 (82.8)	12 (54.5)	0.03^*^
<20/200	6 (17.1)	10 (45.6)	
TR stage	36	20	
<Stage III	23 (63.8)	5 (25)	0.01^*^
≥Stage III	14 (38.9)	15 (75)	
Local therapy			
Any	5 (13.5)	6 (24)	0.33
Photocoagulation	5 (13.5)	6 (24)	0.33
Intravitreal anti-VEGF	2 (5.4)	0	0.51
Cataract surgery	0	2 (8)	0.16
Anti-glaucoma therapy	2 (5.4)	0	0.51

Data are presented as median [interquartile range] or number (percentage).

TA, Takayasu’s arteritis; TR, Takayasu’s retinopathy; MP, methylprednisolone; VEGF, vascular endothelial growth factor.

a Statistical analysis was only made in eye-level comparisons, as the sample size in patient-level analysis (n = 7 in the non-improvement group) was too small for reliable statistical comparisons.

b Collateral formation was available in 24 patients.

c Initial visual acuity was available in 57 patients.

^*^p < 0.05, which indicated statistical differences between groups.

**Figure 7:**
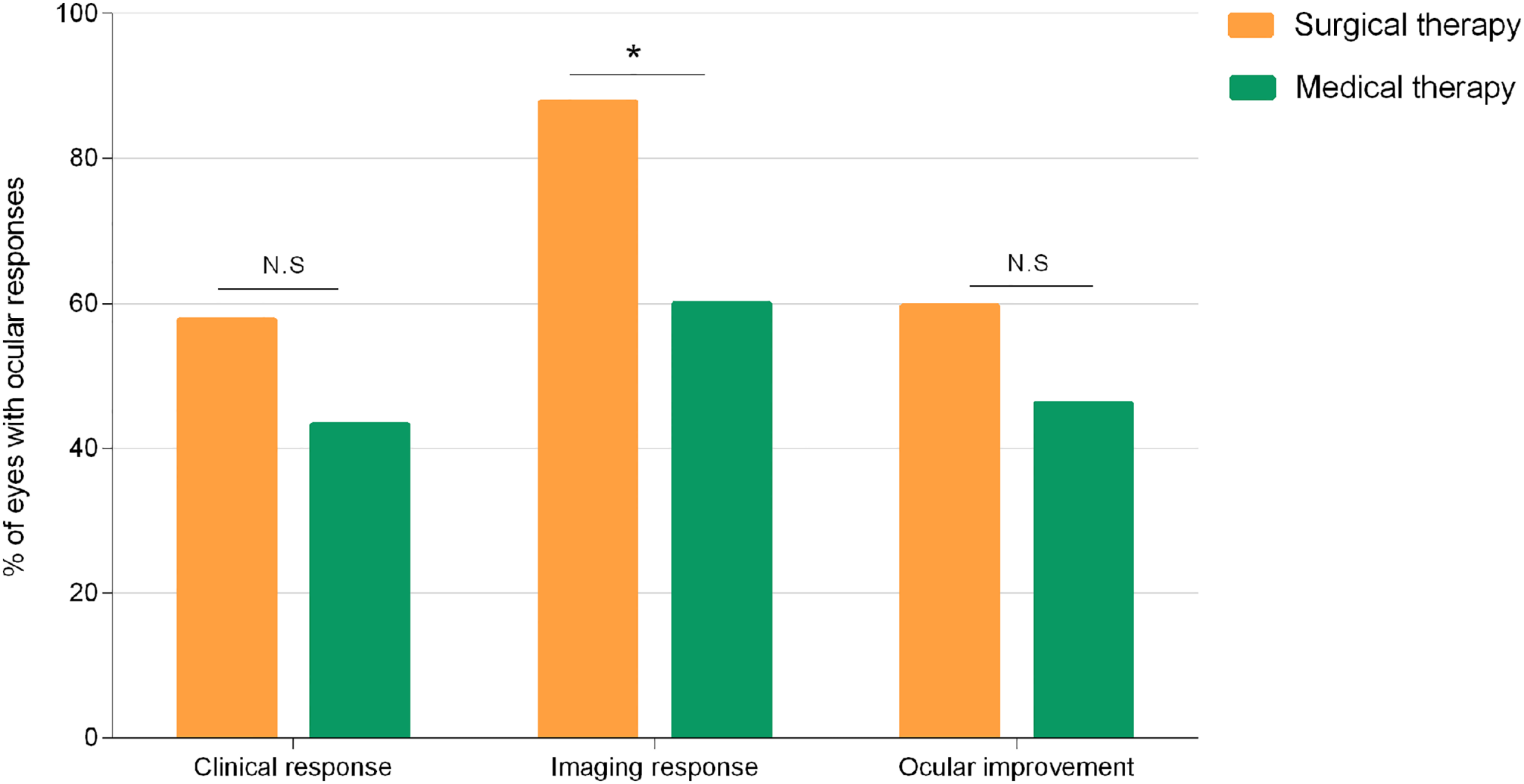
Percentage of eyes with clinical response, imaging response, and overall ocular improvement observed in medical and surgical groups. Comparisons made with chi-squared test between groups. *p < 0.05. N.S., not significant.

Eye-level comparisons of ocular responses between TR patients treated with surgery and medication alone are depicted in **Figure 7**. Surgery is overall more effective considering ocular responses in TR patients. Of note, surgery-treated patients experienced significantly better imaging responses than patients treated with medication alone (p = 0.03).

### Complications

In the medical group, ocular complications were presented in 9 eyes (17.6%) of 6 patients: four cataracts (53, 58, 61), three NVG (47, 54), two central RAO (CRAO) (61), one phthisis bulbi (53), and one steroid-related glaucoma (61). One patient with bilateral cataract underwent cataract surgery in the left eye, where phthisis bulbi developed subsequently. In two patients complicated with NVG, anti-glaucoma drops and panretinal photocoagulation (PRP) were performed with unknown efficacy. In the case complicated with CRAO, intravenous pulse MP and infliximab were added. However, optic atrophy developed as irreversible damages had occurred.

Postoperative ocular complications were described in 7 eyes (10.6%) of 5 patients: four NVG (20, 28, 34), two phthisis bulbi (20), one cataract (28), and one branch RAO (BRAO) (20). Of note, all but one eye with BRAO had TR stage III or above. NVG was demonstrated to be successfully treated with antiglaucoma medication (systemic acetazolamide and topical timolol 0.5%), PRP, and intravitreal anti-VEGF drugs in 3 eyes. One eye developing NVG and concomitant cataract underwent combined surgery. No intervention was possible for eyes with other complications.

Two patients died in the medical group: one due to a cardiac accident and the other due to a cerebrovascular event (49, 58). In the surgical group, one modality was observed during bypass grafting surgery due to severe carotid steal syndrome (31). Restenosis occurred in 2 cases: in-stent restenosis (ISR) in one case (59) and graft narrowing in the other (19). A vascular event due to embolism from diseased vessels was reported 6 months after stenting in one case (29). For the ISR patient, her good vision started to deteriorate 6 months postoperatively when ISR occurred, which improved again after repeated angioplasty. The other two patients, however, experienced varying degrees of ocular improvement despite systemic setbacks.

## Discussion

While steroids and IS represent the mainstay treatment for TA, surgical intervention should be considered for uncontrolled TA, especially those with progressive ocular ischemia refractory to medication (38). Here, we described a dramatic resolution of retinopathy following balloon angioplasty in a patient with TA. This case highlights the efficacy of surgical therapy for ocular ischemia and the novel role of OCTA in perioperative monitoring. A systematic review was also conducted to address the therapeutic management of TA on ocular outcomes.

In this review, the median age at ocular diagnosis was 27 years, similar to 24–41.8 years in the previous cohorts reporting ocular findings of TA (3, 5, 20, 63). Most cases were seen in Asian women. Patients with ocular ischemia were more likely to suffer progressive visual loss concomitant with cerebrovascular signs, such as syncope or stroke. A detailed inquiry into neurologic symptoms may in turn alert rheumatologists for ocular examinations, as vision loss in TA can be insidious or temporary. TR, especially those at stage II, was the most common finding in TA patients. Physicians should thus bear in mind that a routine FFA should be conducted in suspected patients since fundus image might be completely normal at this stage. Since visual symptoms can be presenting manifestations observed in 4 cases, ophthalmologists should also recall this life-threatening disease particularly in young women. Collateral formation, which is commonly established in TA, was observed in 15.5% of the patients. Two collateral pathways have been reported to compensate for the progressive stenosis of the carotid artery (64, 65). While 8 cases developed a major collateral pathway through the circle of Willis, one case had the ophthalmic artery recruited, leading to reversed blood flow and aggravated retinal ischemia. The most common surgery performed was aorto-carotid bypass grafting, probably due to the presence of longer and more fibrotic vessels in these patients (12). PRP was the most frequently performed local therapy to ablate the ischemic peripheral retina in advanced stages of TR.

When evaluating therapeutic outcomes of TA patients with ocular ischemia, clinicians should be aware of discrepancies between clinical and imaging responses, as regression of retinopathy on FFA was more frequently achieved especially in the surgical groups. Chun et al. (3) reported poor final BCVA in two cases in spite of improved retinal circulation after bypass grafting surgery. A similar discrepancy was observed in patients who underwent endovascular procedures as well (20). One explanation could be the occurrence of postoperative complications. Dogra et al. (20) reported a case of TR who had a complete reversal of retinopathy on FFA and experienced a visual worsening in one eye due to the development of BRAO. The choice of imaging modalities might be another issue of concern. Previous literature proposed relatively well-maintained perfusion in the central retina of TA patients (3). However, the theory has been recently challenged with the advent of OCTA. Chotard et al. (22) analyzed 14 eyes of 7 patients with TA who underwent OCTA. A significant correlation was described between foveal avascular zone enlargement on OCTA and decreased BCVA. More so, those microvascular macular abnormalities were even observed in earlier stages and seemed to be correlated with TR severity. To date, only two cases with stage IV TR provided longitudinal changes on OCTA at optic nerve head after systemic therapy (21, 41). In our described case, a follow-up OCTA showed recovery of macular vasculature after balloon angioplasty, paralleling her gradual visual improvement. In one case, ocular blood flow detected by CDI improved 10 days after surgery, followed by delayed recovery of retinal function in electroretinogram at 3 months (32). Taken together, there are chances that macular ischemia and impaired retinal function may persist in spite of resolved peripheral lesions on FFA, resulting in poor visual outcomes. Thus, the abovementioned imaging modalities should be integrated as a complement to FFA for ocular surveillance in TA. It is also anticipated that ultra-wide-field OCTA might be a future alternative to FFA especially for perioperative monitoring, in terms of its non-invasive nature and better visualization of retinal microvasculature recovery (66).

Interestingly, our patient had a dramatic resolution of retinopathy after surgery, whereas little regression of radiological lesions was found. A similar trend was also noticed in 3 cases in the literature, all of which displayed sustained ocular improvement in spite of persistent vascular inflammation, postsurgical embolism, and graft narrowing (19, 29, 40). Possible explanations could be the lack of standardized imaging for follow-up interpretations and the progressive nature of ocular ischemia. Physicians should thus pay more attention to the follow-up ocular examinations in this particular cohort, as they might be complementary or even alternative methods to evaluate therapeutic outcomes of TA.

Bilateral carotid reconstruction should be carefully considered in TA patients, especially for those with preoperative ocular hypertension, to avoid sudden and massive aqueous production (15, 20). Our findings suggest a potential effect of unilateral carotid reconstruction on the contralateral ocular outcome. A possible explanation could be the initiation of perioperative medications (67). In addition, established patency on one side may reduce the collateral demand from the other, restoring contralateral ocular perfusion, as indicated in cerebral revascularization (68, 69).

When comparing patients with and without ocular improvements, statistically significant conclusions on patient-level ocular outcomes are difficult to make because of limited data in the literature. However, our study still highlights the neglected importance of systemic treatment on this particular cohort. A consensus on corticosteroids use has been established in TA management. Adjunctive IS, such as methotrexate and azathioprine, is recommended at the diagnosis of TA (70). Corticosteroids, even administered alone, seem to be effective in ameliorating retinal ischemia, especially when combined with surgical and local therapies in advanced eyes. Unlike daily doses of prednisone, intravenous pulse MP was mostly reserved for patients with severe retinopathy or other ischemic complications (40, 47, 51, 71). Although methotrexate remains the first IS choice of many physicians due to its inexpensive and relatively safe nature (72), its efficacy on ocular ischemia might be less than expected for an unknown reason. An alternative IS, such as CTX, can thus be considered when visual symptoms continue to progress (73, 74). Noel et al. proposed IS should be promptly added to limit functional sequelae in TA with acute retinal ischemia (61). However, evidence is not conclusive in our study as to under which circumstances IS should be combined in the main cohort. Antiplatelet agents have been proven to reduce ischemic events and the risk of restenosis in TA (1, 75). One-third of included patients were treated with aspirin and/or clopidogrel, with slightly better ocular outcomes observed in both medication and surgery groups, consistent with previous studies (75, 76). Biological agents, such as infliximab, should be discussed in acute retinal ischemia when other therapies have failed (61). Overall, we recommend a prompt initiation of oral steroids, in the combination of IS and antiplatelet agents in patients with ocular ischemia, especially for those showing contradictions or unwillingness to surgery. Visual progression should be closely monitored for the necessity of IS switch and biological agents.

As is known, bypass grafting is superior to endovascular therapy in terms of long-term patency (15, 77), but it is associated with a higher rate of stroke when supra-aortic branches are involved (78). To date, no comparative studies exist pertaining to the efficacy of these two procedures on ocular outcomes in TA. The present review demonstrated similar rates of visual improvement for open and endovascular procedures at short-term (6 months) follow-up. However, long-term outcomes may differ, as postoperative complications (i.e., restenosis) are more likely to occur over 1 year (15, 79). In literature, visual acuity is usually well-maintained in TA patients with arterial collateralization (80). A previous study suggested a better visual prognosis after bypass grafting in patients with collateral vessels (3). We detected a similar trend in this review with no significant difference, probably due to the limited sample data. Medical therapy given as a perioperative adjunct is rarely documented in the surgery-treated group since most articles were extracted from ophthalmology journals. In the small subgroup of available patients, oral steroids and antiplatelet therapy are particularly essential to postsurgical ocular improvement. Although its efficacy on ocular ischemia is inconclusive based on our review, postsurgical IS treatment should still be recommended for systemic regression (1). Ocular improvement was found to significantly correlate with a higher baseline visual acuity, specifically with 20/200 and above, and a less severe stage of TR (stage I or II). As expected, poor visual acuity most likely implies a greater degree of ischemic insult and thus a more advanced stage of retinopathy (3). These findings are consistent with previous studies (3, 13, 18, 20), indicating that surgical intervention is best recommended before the onset of irreversible ischemia to the globe. Of note, ocular improvement is observed even in cases with stage IV TR, as long as the surgery is timely performed and adjunctive PRP is planned (19, 20, 37).

When it comes to the optimal choice of therapy, the present review suggests an overall superiority of surgical treatment to medical therapy alone especially regarding imaging responses in TR. The result is comparatively reliable since no significant differences in clinical parameters were found between groups. Acute retinal ischemia has been largely overlooked in TA patients, as they occurred very rarely (61). Tyagi et al. (59) suggested that acute vision loss, such as AION and RAO, may not be amendable to the restoration of retinal blood flow. However, sporadic case reports have challenged the opinion (8, 21, 25). In the present review, carotid surgery is superior to medical therapy in these patients in terms of visual improvement. Altogether, we speculate that both acute and chronic vision loss can be better controlled by surgical therapy in patients with TA. However, we still highly recommend a detailed discussion with a multidisciplinary team (including rheumatologists, vascular surgeons, ophthalmologists, and radiologists) for individual management.

Compared with medical therapy, surgery is also a better choice in terms of lower complications. Surgical treatment is safe overall in TA patients with ocular ischemia, as mortality occurred in only one case. Cerebrovascular or cardiac accidents should be cautioned in patients treated with medical therapy alone. NVG occurs as a failure of therapies, preferentially in patients with prolonged ocular ischemia, advanced stages of TR, and preexisting iris rubeosis (34, 54). In surgery-related NVG, surgical intervention can quickly restore circulation to the ischemic area of the globe, resulting in massive raise in aqueous production and subsequent intraocular hypertension. In addition, the preexisting new vessels in the anterior chamber angle may cause additional obstruction of the meshwork, thereby more prone to develop NVG (59, 81). These patients may sometimes present with normal or decreased IOP, due to chronic ischemia of the ciliary body (81). Patients should thus be carefully monitored for visual symptoms and IOP after the procedure to accurately diagnose and treat NVG. A concomitant cataract may occur in patients with NVG, supporting the idea that exaggerated ocular ischemia, with elevated vascular endothelial growth factor levels, can result in metabolic changes of the lens (28, 82). Cataract surgery needs to be performed with caution in the presence of ocular ischemia, as disruption of the balance between ocular perfusion and intraocular inflammation might occur, resulting in phthisis bulbi (53). Phthisis bulbi that occurred after surgery was probably due to irreversible endothelial damage from ischemia–reperfusion injury and secondary shutdown of aqueous production (20). RAO is a rare complication after therapy. An anatomical similarity between the retinal arteries and vasa vasorum, where the inflammation initially presents, may play a role (61). Besides, emboli originating from the internal carotid artery during wiring and stent passage may also be responsible (83).

The described case highlighted the efficacy of balloon angioplasty on ocular outcomes, along with the importance of OCTA in postoperative management in TA patients with ocular ischemia. By conducting this never-performed review, we provided uniform data and future therapeutic guidance of this particular cohort for clinicians. However, several limitations of the present systematic review should be addressed. First, all studies included in this review are case reports or retrospective case series, limiting the use of meta-analysis. The possibility of publication bias should not be neglected as well. Second, the sample size of our study is small with a short follow-up period, resulting in a lack of power to detect certain significant findings. Results and conclusions are mostly of descriptive nature and should therefore be interpreted with caution. Considering the rarity of the disease, our data involving 66 patients are sufficient for clinical guidance in these patients. Third, data regarding perioperative medications are rather limited, which needs to be addressed in future studies with larger population size and longer follow-up.

## Conclusion

Clinicians should be familiar with ophthalmic manifestations of this potentially treatable complication in TA. Although medical therapy alone seems to be effective for TR, surgical intervention in combination with perioperative medications and local therapy might be a better choice for both acute and chronic vision loss. Medications alone might be effective for those with surgical contradictions or reluctance to an invasive procedure. Surgery is best recommended when performed before the onset of irreversible ischemia to the globe. However, no short-term superiority of open surgery versus endovascular procedure was found in terms of ocular outcomes. Physicians should be aware of the importance of ocular examinations, especially FFA and OCTA, during the diagnosis and follow-up in TA. Studies with a larger population size and longer follow-up should be encouraged to better address this issue.

## Data Availability Statement

The raw data supporting the conclusions of this article will be made available by the authors, without undue reservation.

## Author Contributions

MZ conceived and designed the study. YZ, JD, and MZ were involved in clinical management. YZ, JD, and GG contributed to literature search, data acquisition, and data analysis for the systematic review. YZ drafted and revised the paper. JD contributed to all the figures. All authors provided critical feedback and approved the submitted version.

## Funding

This work is supported by 1.3.5 project for disciplines of excellence, West China Hospital, Sichuan University (ZYJC21025).

## Conflict of Interest

The authors declare that the research was conducted in the absence of any commercial or financial relationships that could be construed as a potential conflict of interest.

## Publisher’s Note

All claims expressed in this article are solely those of the authors and do not necessarily represent those of their affiliated organizations, or those of the publisher, the editors and the reviewers. Any product that may be evaluated in this article, or claim that may be made by its manufacturer, is not guaranteed or endorsed by the publisher.
