# Supplementary material for: Therapeutic Management of Ocular Ischemia in Takayasu’s Arteritis: A Case-Based Systematic Review
Source: Front Immunol. 2022 Jan 14;12:791278. doi: 10.3389/fimmu.2021.791278 (PMC8795594; doi:10.3389/fimmu.2021.791278)
Supplement: Supplementary file 1 [file Table_1.docx]

Supplemental Table 1. Methodological quality and synthesis of case series and case reports using modified Newcastle-Ottawa scale.

| Author, year |  | Selection | Ascertainment | | Causality | Reporting | Methodological quality |
| --- | --- | --- | --- | --- | --- | --- | --- |
|  |  | Does the patient(s) represent(s) the whole experience of the investigator (center) or is the selection method unclear to the extent  that other patients with similar presentation may not have been reported? | Was the exposure adequately ascertained? | Was the outcome adequately ascertained? | Was follow-up long enough for outcomes to occur? | Is the case(s) described with sufficient details to allow other investigators to replicate the research or to allow practitioners make  inferences related to their own practice? |  |
| Reddy et al 2020 |  | NO | YES | YES | YES | YES | Moderate |
| Rullo et al 2019 |  | NO | YES | YES | YES | NO | Poor |
| Dogra et al 2019 |  | YES | YES | YES | YES | YES | Good |
| Christiansen et al 2019 |  | NO | YES | YES | YES | YES | Moderate |
| Tyagi et al 2018 |  | NO | YES | NO | YES | NO | Poor |
| Tian et al 2018 |  | NO | YES | YES | YES | YES | Moderate |
| Subira et al 2018 |  | NO | YES | YES | YES | YES | Moderate |
| Bajgai et al 2018 |  | NO | YES | YES | YES | YES | Moderate |
| Lee et al 2014 |  | NO | YES | YES | YES | YES | Moderate |
| Goldman et al 2013 |  | NO | YES | YES | YES | YES | Moderate |
| Peter et al 2013 |  | NO | YES | YES | YES | YES | Moderate |
| Xiang et al 2013 |  | NO | NO | YES | YES | NO | Poor |
| Wang et al 2012 |  | YES | YES | YES | NO | YES | Moderate |
| Peter et al 2010 |  | NO | YES | YES | YES | YES | Moderate |
| Koz et al 2007 |  | NO | YES | YES | YES | YES | Moderate |
| Vedantham et al 2005 |  | NO | YES | YES | YES | YES | Moderate |
| Kinoshita et al 2005 |  | NO | YES | YES | YES | YES | Moderate |
| Slusher et al 2002 |  | NO | YES | YES | YES | YES | Moderate |
| Chun et al 2001 |  | YES | YES | YES | YES | YES | Good |
| Author, year |  | Selection | Ascertainment | | Causality | Reporting | Methodological quality |
|  |  | Does the patient(s) represent(s) the whole experience of the investigator (center) or is the selection method unclear to the extent  that other patients with similar presentation may not have been reported? | Was the exposure adequately ascertained? | Was the outcome adequately ascertained? | Was follow-up long enough for outcomes to occur? | Is the case(s) described with sufficient details to allow other investigators to replicate the research or to allow practitioners make  inferences related to their own practice? |  |
| Kapran et al 2001 |  | NO | YES | YES | YES | YES | Moderate |
| Milea et al 1999 |  | NO | YES | YES | NO | YES | Poor |
| Karam et al 1999 |  | NO | YES | YES | NO | YES | Poor |
| Lewis et al 1993 |  | NO | YES | YES | YES | YES | Moderate |
| Ueno et al 1967 |  | NO | YES | YES | YES | YES | Moderate |
| Austen et al 1964 |  | NO | YES | YES | YES | YES | Moderate |
| Rahman et al 2021 |  | NO | YES | YES | YES | YES | Moderate |
| Surya et al 2020 |  | YES | YES | NO | NO | YES | Poor |
| Larrazabal et al 2020 |  | YES | YES | NO | YES | YES | Moderate |
| Gong et al 2020 |  | YES | YES | YES | YES | YES | Good |
| Kannan et al 2019 |  | YES | YES | YES | YES | YES | Good |
| Anguita et al 2019 |  | YES | YES | YES | YES | YES | Good |
| Pallangyo et al 2010 |  | YES | YES | NO | YES | YES | Moderate |
| Gaur et al 2017 |  | YES | YES | YES | YES | NO | Moderate |
| Mashru et al 2016 |  | NO | YES | YES | YES | YES | Moderate |
| Guclu et al 2016 |  | NO | YES | YES | YES | YES | Moderate |
| Santhanam et al 2015 |  | YES | YES | NO | YES | YES | Moderate |
| Matsumoto-Otake et al 2015 |  | YES | YES | YES | YES | YES | Good |
| Malik et al, 2015 |  | YES | YES | NO | YES | YES | Moderate |
| Shailaja et al 2013 |  | YES | YES | NO | YES | YES | Moderate |
| Author, year |  | Selection | Ascertainment | Causality | Causality | Reporting | Methodological quality |
|  |  | Does the patient(s) represent(s) the whole experience of the investigator (center) or is the selection method unclear to the extent  that other patients with similar presentation may not have been reported? | Was the exposure adequately ascertained? | Was the outcome adequately ascertained? | Was follow-up long enough for outcomes to occur? | Is the case(s) described with sufficient details to allow other investigators to replicate the research or to allow practitioners make  inferences related to their own practice? |  |
| Noel et al 2013 |  | NO | YES | YES | YES | YES | Moderate |
| Sakthiswary et al 2012 |  | YES | YES | YES | YES | YES | Good |
| Pelegrin et al 2012 |  | YES | YES | YES | YES | YES | Good |
| Wang et al 2012 |  | YES | NO | YES | YES | YES | Moderate |
| Demir et al 2010 |  | YES | YES | NO | YES | YES | Moderate |
| Das et al 2010 |  | NO | YES | YES | YES | YES | Moderate |
| Kaushik et al 2005 |  | NO | YES | YES | YES | YES | Moderate |
| Park et al 1999 |  | NO | YES | YES | YES | YES | Moderate |
| Karam et al 1999 |  | YES | NO | NO | NO | YES | Poor |
| Schmidt et al 1997 |  | NO | YES | YES | YES | YES | Moderate |
| Paterson et al 1957 |  | YES | NO | YES | YES | NO | Poor |
