# Supplementary material for: Therapeutic Management of Ocular Ischemia in Takayasu’s Arteritis: A Case-Based Systematic Review
Source: Front Immunol. 2022 Jan 14;12:791278. doi: 10.3389/fimmu.2021.791278 (PMC8795594; doi:10.3389/fimmu.2021.791278)
Supplement: Supplementary file 2 [file Table_2.docx]

Supplementary Table 2. Characteristics of studies that reported visual outcomes after surgical intervention in TA patient.

| Author, year | Age/  sex | Initial visual acuity | Ocular examination | Diagnosis | Surgical procedure | Collateral vessels | Medical treatment | Ocular procedure | Follow-up (week) | Post-operation  outcome | | Complication |
| --- | --- | --- | --- | --- | --- | --- | --- | --- | --- | --- | --- | --- |
| Reddy et al 2020 | 27/F | OD 20/100  OS 20/125 | OU delayed retinal circulation, MA, NVD, capillary non-perfusion | OU TR stage IV  OU AION | Bilateral carotid endarterectomy | NA | NA | OU PRP | 8 | OD 20/40  OS 20/60  OU optic disc pallor, regressed NVD | | NONE |
| Rullo et al 2019 | 57/F | OD 20/30  OS 20/20 | OD large retinal pallor with mild disc elevation, OS white-centered hemorrhage | Type 1 TA  OD ischemic retinopathy (BRAO) | Bypass procedure | NO | - oral steroids | NONE | 8 | OD improved vision and central visual field defect | | NONE |
| Dogra et al 2019 | 18/F | OD HM  OS HM | OD cotton wool spots, OU dilated veins, MA, AV neovascularization of the disc | OU TR stage IV | Right CCA + VA stenting followed by left VA stenting after 3 months | NA | NA | NONE | 312 | OD 20/200  OS NLP  OD TR regression to stage I, OS remained stage IV | | OD optic atrophy, OS phthisis bulbi |
|  | 20/F | OD NLP  OS HM | OU sluggish retinal flow MA, optic disc pallor | Type 1 TA  OU TR stage IV | Left CCA +VA stenting followed by right CCA + VA stenting after 3 months | NO | - oral steroids | NONE | 58 | OD NLP  OS 20/400  OD remained stage IV, OS normalization of blood flow, less dilated vein, MA disappearance | | OD phthisis bulbi, OS optic atrophy |
|  | 29/F | OD 20/20  OS 20/60 | NA | OU TR stage II | Left CCA +VA  stenting followed by right CCA stenting after 1 month | NA | NA | NONE | 78 | OD 20/20  OS 20/80  OU complete reversal of TR | | OS BRAO |
|  | 28/F | OD 20/120  OS 20/120 | NA | OU TR stage II | Right CCA stenting followed by left CCA + VA stenting after 1 month | NA | NA | NONE | 48 | OD 20/20  OS 20/20  OU complete reversal of TR | | NONE |
|  | 35/F | OD 20/80  OS 20/120 | OU MA, AV, capillary non-perfusion | OU TR stage III | Right CCA + VA stenting followed by left VA stenting after 1 month | NA | NA | OU PRP + intravitreal anti-VEGF +antiglaucoma drugs | 26 | OD 20/60  OS 20/80  OD decreased MA;  OU progressed to stage IV | | OU NVG |
| Author, year | **Age/**  **sex** | **Pre-operation visual acuity** | **Ocular examination** | **Diagnosis** | **Surgical procedure** | **Collateral vessels** | **Medical treatment** | **Ocular procedure** | **Follow-up (week)** | **Post-operation**  **ocular outcome** | | **Complication** |
| Christiansen et al 2019 | 19/F | OD 20/200  OS 20/25 | OD retinal and optic disc pallor, arteriolar narrowing, OU sluggish retinal flow | Type 2a TA  OU ischemic retinopathy (TR) | Bilateral CCA bypass grafting | YES | - Intravenous methylprednisolone 1000 mg for 3d  - high-dose oral prednisone  - methotrexate  - infliximab  - dual antiplatelet | NONE | 24 | OD no vision recovery, OS resolved amaurosis, restoration of anterograde flow in the right ophthalmic artery | | NONE |
| Tyagi et al 2018 | 23/M | NA | OD disc edema, cherry spot at macula | Type 1 TA  OD CRAO | Pre-vertebral right subclavian artery stenting | NO | - oral steroids  - aspirin 150mg/d  - clopidogrel 75mg/d | NONE | 96 | OD good improvement in vision | | NONE |
|  | 27/F | NA | OS optic disc pallor | Type 1 TA  OS ischemic retinopathy (TR) | Left SCA balloon angioplasty+ VA drug-eluting stent, followed by left CCA drug-eluting stent | NO | - oral steroids  - antiplatelet  - methotrexate | NONE | 24 | OS marked improvement in visual symptoms for 6 months, OS vison started to deteriorate | | severe in-stent restenosis, cutting balloon angioplasty was done, OS vision improved |
| Tian et al 2018 | 23/F | OD 20/400  OS 20/25 | OD optic disc pallor, MA; OS optic disc edema with late dye leakage | Type 2b TA  OU AION | Balloon angioplasty of bilateral CCA | NO | NA | NONE | 12 | OD 20/40  OS 20/20; OD optic disc pallor, OS resolution of optic disc edema | | NONE |
| Subira et al 2018 | 33/F | OD 20/400 | OD MA, non-perfusion, hemorrhages, disc neovascularization | Type 1 TA  OD TR stage IV | aorta to right carotid bypass grafting | NO | NA | OU PRP | 3 | OD ocular pain, IOP 42 mmHg, NVG, total white cataract | | OD cataract surgery and anti-glaucoma surgery |
| Bajgai et al 2018 | 25/F | OD LP  OS HM | Impaired retinal circulation, retinal boxcarring | OU TR stage II | CCA+VA stenting | NA | - oral steroids  - azathioprine | NONE | 4 | OD LP  OS 20/400 | | NONE |
| Lee et al 2014 | 28/F | OD 20/20  OS 20/20 | OU mildly attenuated retinal vessels, white- centered hemorrhages, MA | Type 2b TA  OU TR stage II | Ascending aorta to the right/left SCA bypass grafting | NO | NA | NONE | 8 | Resolved ocular symptoms, resolution of hemorrhages | | NONE |
| Goldman et al 2013 | 32/F | NA | OU delayed retinal circulation, MA, capillary non-perfusion | OU TR stage II | surgical revascularization | NA | NA | NONE | 20 | OU resolution of capillary ischemia and leakage, a few MA left | | NONE |
| Author, year | **Age/**  **sex** | **Pre-operation visual acuity** | **Ocular examination** | **Diagnosis** | **Surgical procedure** | **Collateral vessels** | **Medical treatment** | **Ocular procedure** | **Follow-up (week)** | **Post-operation**  **ocular outcome** | | **Complication** |
| Peter et al 2013 | 37/F | OD 20/120  OS NLP | OD mild disc pallor, MA; OS optic atrophy, MA | Type 1 TA  OU TR stage II | Right CCA + SCA stenting followed by left SCA stenting 6 weeks later | NO | - oral steroids  - mycophenolate  - aspirin and clopidogrel | NONE | 24 | OD 20/50  OS LP;  OU regression of MA | | A vascular event due to embolism from diseased proximal vessels |
| Xiang et al 2013 | 16/F | OS 20/40 | OS retinal pallor, OU hyperemic disc | Type 1 TA  OS BRAO | Thoracic aorta to CCA bypass grafting | NO | NA | NONE | 48 | OS 20/25 | | NONE |
| Wang et al 2012 | 25/F | OD 20/16  OS 20/16 | OD MA, OS dilated retinal veins | Type 1 TA  OD TR stage II  OS TR stage I | CCA to SCA bypass grafting | NO | - oral steroids  - oral cyclophosphamide  - aspirin | NONE | 3 days | OU no change | | NONE |
|  | 49/F | OD 20/200  OS 20/200 | OU delayed retinal circulation, MA, cotton wool spots, capillary non-perfusion | Type 1 TA  OU TR stage II | Ascending aorta to CCA bypass grafting | NO | - oral steroids  - oral cyclophosphamide  - aspirin | NONE | 12 | OD 20/100  OS 20/200  Regressed cotton wool spots | | NONE |
|  | 25/F | OD CF  OS CF | OU AV | Type 1 TA  OU TR stage III | Ascending aorta to CCA bypass grafting | NO | - oral steroids  - oral cyclophosphamide  - methotrexate  - aspirin | OU topical steroids, OU PRP | Right after discharge | OU vision unchanged | | NONE |
| Peter et al 2010 | 29/F | OD 20/20  OS HM | OS disc hypo- fluorescence due to loss of perfusion | Type 1 TA  OS AION | CCA+SCA stenting | NO | NA | NONE | 144 | OS vision  unchanged | | NONE |
| Koz et al 2007 | 45/M | OD 20/40  OS 20/40 | OU delayed retinal circulation, MA, capillary non-perfusion, optic disc neovascularization | Type 1 TA  OU TR stage IV | Bypass surgery | YES | - oral prednisolone 1 mg/kg/d  - methotrexate 15 mg/week, replaced by 1 g cyclophosphamide pulse therapy once a month | NONE | Died during bypass surgery | | | |
| Vedantham et al 2005 | 40/F | OD 20/20  OS 20/200 | OU delayed retinal circulation, MA, retinal boxcarring, AV, formation of new vessels | Type 1 TA  OU TR stage IV | Right CCA-SCA bypass grafting | NA | - oral steroids | OU PRP | 36 | OD 20/20  OS 20/40;  OU new vessels regressed, delayed retinal circulation;  OS persisted MA, AV | Graft narrowing | |
| Author, year | **Age/**  **sex** | **Pre-operation visual acuity** | **Ocular examination** | **Diagnosis** | **Surgical procedure** | **Collateral vessels** | **Medical treatment** | **Ocular procedure** | **Follow-up (week)** | **Post-operation**  **ocular outcome** | | **Complication** |
| Kinoshita et al 2005 | 28/F | OD 20/10  OS 20/15 | OU delayed retinal circulation, MA | Type 1 TA  OU TR stage II | Right axillary artery to common iliac artery bypass grafting | NO | NA | NONE | 12 | OU decreased MA, persistent dye leakage, ERG oscillatory potentials detected | | NONE |
| Slusher et al 2002 | 25/F | OD 20/30  OS 20/60 | OS MA, venous engorgement, disc neovascularization | Type 1 TA  OU TR stage III | Bilateral aorta to CCA bypass grafting | NO | - oral prednisone | NONE | 4 | OD 20/25  OS 20/30  OS resolved MA, neovascularization | | NONE |
| Chun et al 2001 | 31/F | OD 20/25  OS 20/20 | OU delayed retinal circulation, vessel dilatation, OS MA | Type 1 TA  OD TR stage I  OS TR stage II | aortic–internal carotid artery bypass grafting | YES | NA | NONE | NA | OU retinal circulation improved | | NONE |
|  | 32/M | OD 20/20  OS 20/25 | OD delayed retinal circulation, vessel dilatation, MA | Type 5 TA  OD TR stage II | aortic–internal carotid artery bypass grafting | NO | NA | NONE | NA | OD symptoms improved, mild, moderate TR, retinal circulation improved, MA decreased | | NONE |
|  | 37/F | OD 20/20  OS 20/20 | OD delayed retinal circulation, MA | Type 1 TA  OD TR stage II | bypass grafting | NO | NA | NONE | NA | OD good BCVA mild, moderate TR, improved retinal circulation | | NONE |
|  | 18/F | OD 20/22  OS 20/20 | OU delayed retinal circulation, vessel dilatation, MA, AV, non-perfusion area | Type 1 TA  OU TR stage III | bypass grafting | NO | NA | NONE | NA | OU good BCVA mild, moderate TR, improved retinal circulation | | NONE |
|  | 25/F | OD HM  OS CF | OU delayed retinal circulation, vessel dilatation, non-perfusion, MA, AV, NVG | Type 1 TA  OU TR stage IV | bypass grafting | NO | NA | NONE | NA | OU poor BCVA, decreased incidence of syncope and amaurosis fugax, improved retinal circulation | | NONE |
|  | 38/F | OD 20/200  OS HM | OU delayed retinal circulation, vessel dilatation, non-perfusion, MA, AV, NVG, NVD, TRD | Type 1 TA  OU TR stage IV | bypass grafting | NO | NA | NONE | NA | OU poor BCVA, decreased syncope and amaurosis fugax incidence, improved retinal circulation | | NONE |
| Author, year | **Age/**  **sex** | **Pre-operation visual acuity** | **Ocular examination** | **Diagnosis** | **Surgical procedure** | **Collateral vessels** | **Medical treatment** | **Ocular procedure** | **Follow-up (week)** | **Post-operation**  **ocular outcome** | | **Complication** |
| Milea et al 1999 | 32/F | OD LP  OS 20/20 | OD cataract, preretinal new vessels, NVG;  OS dilated vessels, MA, AV | Type 1 TA  OU TR stage IV | Right aorto-carotid bypass grafting | NO | - oral prednisolone 30 mg/d  - aspirin 100mg qd | OU PRP  OD cataract surgery | 6 hours | red painful right eye, OD IOP 40 mmHg, closed right angle, leakage from the right prepapillary vessels; OS unchanged | | OD NVG |
| Karam et al 1999 | 24/F | OD 20/30  OS 20/30 | OU delayed retinal circulation, MA, non-perfusion, AV | Type 1 TA  OU TR stage III | Bilateral aorto-carotid artery bypass grafting | YES | NA | NONE | NA | OD 20/20  OS 20/20  OU regressed MA, persistent capillary non-perfusion | | NONE |
| Lewis et al 1993 | 59/F | OD HM  OS 20/200 | OU delayed retinal circulation, MA, dot hemorrhages, pale discs, OS RNFL infarcts | Type 1 TA  OU TR stage II | Bypass grafting | YES | - intravenous methylprednisolone 250 mg q6h  -intravenous heparin  - oral steroids  - aspirin 325mg | NONE | 20 | OD 1/200  OS 20/50  OD pale disc, OU restored circulation, resolved MA, hemorrhages and RNFL infarcts | | NONE |
| Ueno et al 1967 | 17/F | OD 15/20  OS 15/20 | OU delayed retinal circulation, MA, hemorrhages | Type 2b TA  OU TR stage II | Left aorto-carotid bypass grafting | NO | NA | NONE | 3 | Normal OU IOP, Normalized retinal blood flow, regressed MA and hemorrhages | | NONE |
| Austen et al 1964 | 28/F | OD NLP  OS 20/20 | OU pale fundi, AV, neovascularization | Type 1 TA  OU TR stage IV | Bilateral aorto-carotid bypass grafting | YES | NA | NONE | 12 | OD HM  OS 20/20 | | NONE |
| Our case | 29/F | OD 20/1000  OS 20/50 | OU delayed retinal circulation, MA | Type 5 TA  OU TR stage II | Bilateral SCA balloon angioplasty | NO | - intravenous cyclophosphamide once a month  - oral prednisone 40mg/d  - cyclophosphamide 50mg/d  - aspirin  - vasodilators | NONE | 24 | OD 20/30  OS 20/50  OU Normalized retinal blood flow, regressed MA | | NONE |

TA: Takayasu’s arteritis; TR: Takayasu’s retinopathy; MA: microaneurysms; AV: arteriovenous shunts; NVG: neovascular glaucoma; NVD: neovascularization of optic nerve head; TRD: tractional retinal detachment; PRP: pan retinal photocoagulation; CCA: common carotid artery; SCA: subclavian artery; VA: vertebral artery; M: male; F: female; OD: right eye; OS: left eye; OU: both eyes; BCVA: best-corrected visual acuity; CF: count finger; HM: hand movement; LP: light perception; NLP: no light perception; IOP: intraocular pressure; VEGF: vascular endothelial growth factor; ERG: electroretinogram; RNFL: retinal nerve fiber layer; PTA: percutaneous transluminal angioplasty; AION: anterior ischemic optic neuropathy; BRAO: branch retinal artery occlusion; CRAO: central retinal artery occlusion; NA: not applicable.
