# Supplementary material for: Therapeutic Management of Ocular Ischemia in Takayasu’s Arteritis: A Case-Based Systematic Review
Source: Front Immunol. 2022 Jan 14;12:791278. doi: 10.3389/fimmu.2021.791278 (PMC8795594; doi:10.3389/fimmu.2021.791278)
Supplement: Supplementary file 3 [file Table_3.docx]

Supplementary Table 3. Characteristics of studies that reported ocular outcomes after medical therapy alone in TA patient.

| Author, year | Age/  sex | Initial visual acuity | Ocular examination | Diagnosis | Medical therapy | Collateral vessels | Ocular procedure | Follow-up (week) | Ocular outcome | | | | Complication |
| --- | --- | --- | --- | --- | --- | --- | --- | --- | --- | --- | --- | --- | --- |
| Rahman et al 2021 | 22/F | OD 20/30  OS 20/120 | OD venous dilation, MA; OS delayed retinal circulation, capillary non-perfusion | Type 1 TA  OU TR stage II | - six cycles of intravenous cyclophosphamide  - oral prednisone  - azathioprine and methotrexate  - mycofenolate | NO | OU PRP | 48 | OU stable vision, persisted NVD, progressed to stage IV | | | | |
| Surya et al 2020 | 18/F | OD 20/40  OS 20/120 | OD boxcarring vessels, MA; OS delayed retinal circulation, peripheral avascularity, NVD | Type 1 TA  OD TR stage II  OS TR stage III | - methylprednisolone 750 mg pulse IV  - oral steroids 1 mg/kg  - oral azathioprine 25mg | NO | NONE | 4 | OS regressed NVD | | | | NONE |
| Larrazabal et al 2020 | 26/F | OD LP  OS LP | OD diffuse occlusive retinopathy, NVD, macular edema; OS mature cataract | Type 5 TA  OU TR stage IV | - intravenous methylprednisolone 500 mg daily for 3 days  - prednisone 35mg/d  - aspirin 80mg/d | NO | NONE | 8 | OU vision improvement | | | | NONE |
| Gong et al 2020 | 18/F | OD 20/25  OS HM | OD NVD, MA, AV; OS vitreous hemorrhage, retinal detachment | Type 5 TA  OU TR stage IV | - oral prednisone 60mg/d  - leflunomide 20mg/d  - beraprost 60μg/d  - aspirin 100mg/ d | NO | OU intravitreal ranibizumab; OS vitrectomy | 24 | OD 20/20, regressed NVD  OS 20/400, retinal vessels recanalized | | | | NONE |
| Kannan et al 2019 | 13/M | OD 20/20  OS 20/1200 | OD venous dilation, capillary non-perfusion; OS RAPD, NVI, venous dilation, AV, delayed retinal circulation, capillary non-perfusion | Type 1 TA  OD TR stage II  OS TR stage IV | - oral steroids 1mg/kg/d  - oral methotrexate 10 mg/week | YES | OS three doses of intravitreal bevacizumab | 20 | OD no worsening of signs or symptoms  OS NLP, persisted NVI with normal IOP, pale optic disc, attenuated arterioles | | | | NONE |
| Anguita et al 2019 | 12/F | OD 20/20  OS 20/20 | OU venous dilation, MA, cotton wool spots; delayed retinal circulation, capillary non-perfusion | Type 1 TA  OU TR stage II | - oral steroids  - mycofenolate | NO | NONE | NA | OU good perfusion | | | | NONE |
| Author, year | **Age/**  **sex** | **Initial visual acuity** | **Ocular examination** | **Diagnosis** | **Medical therapy** | **Collateral vessels** | **Ocular procedure** | **Follow-up (week)** | **Ocular outcome** | | | | **Complication** |
| Pallangyo et al 2010 | 24/F | OD CF  OS NLP | OU MA, dot hemorrhages, capillary non-perfusion, neovascularization | Type 5 TA  OU TR stage IV | - dexamethasone 60mg/d  - methotrexate 10mg/week  - aspirin 75mg/d | NO | NONE | 12 | OU visual acuity neither improvement nor deterioration | | | | NONE |
| Gaur et al 2017 | 27/F | OD LP  OS 20/120 | OD RAPD; OU delayed circulation, disc pallor, MA | Type 1 TA  OU TR stage II | - oral steorids | NO | NONE | 12 | OD no visual improvement  OS 20/40 | | | | NONE |
| Mashru et al 2016 | 31/F | OD 20/20  OS CF/3m | OS retinal edema | Type 5 TA  OS BRAO | - oral prednisone 1 mg/kg/d | NO | NONE | 8 | OS 20/60  OS healed preretinal hemorrhage and optic atrophy | | | | NONE |
| Guclu et al 2016 | 48/F | OD LP  OS 20/20 | OD RAPD, pale retina with cherry red spot | Type 1 TA  OD CRAO | - intravenous methylprednisolone 4×250mg/kg/d for two days  - followed by oral prednisone 48mg/d  - methotrexate 10mg/week | NO | NONE | 144 | OD NLP, pale optic disc | | | | NONE |
| Santhanam et al 2015 | 19/F | OD LP  OS LP | OU pale disc, dilated veins, MA, capillary non-perfusion | Type 5 TA  OU TR stage II  OU AION | - oral steroids 1mg/kg/d  - methotrexate 15mg/kg  - antiplatelets  - statins, antihypertensives | NO | NONE | 8 | OU no improvement in vision | | | | NONE |
| Matsumoto-Otake et al 2015 | 31/F | OD 20/33  OS 20/100 | OU dilated veins, AV, thickening of the retinal ganglion cell complex; OS vitreous hemorrhage | Type 5 TA  OU TR stage IV | - intravenous pulse methylprednisolone 500mg/d for 3 days  - oral prednisolone  - anti-platelet agents | NO | NONE | 8 | OD 20/50  OS 20/200  OU progression of AV, capillary nonperfusion | | | | OU Rubeosis iridis (followed by OU PRP) |
| Malik et al, 2015 | 35/F | OD HM  OS 20/120 | OU pale disc, venous dilation, sluggish blood flow, | Type 5 TA  OU TR | - dexamethasone 60mg/d | NO | NONE | 12 | OU no improvement in vision | | | | NONE |
| Shailaja et al 2013 | 22/F | OD 20/400  OS 20/400 | OU venous dilatation, delayed circulation MA, dot hemorrhages, cotton wool spots; | Type 2b TA  OU TR stage II | - oral steroids 1mg/kg/d | NO | NONE | 12 | Died of sudden cardiac death | | | | |
| Author, year | **Age/**  **sex** | **Initial visual acuity** | **Ocular examination** | **Diagnosis** | **Medical therapy** | **Collateral vessels** | **Ocular procedure** | **Follow-up (week)** | **Ocular outcome** | **Complication** | | | |
| Noel et al 2013 | 58/F | OD 20/63  OS 20/200 | OU optic disk pallor, bilateral peripheral ischemia | Type 5 TA  OU BRAO | - Intravenous methylprednisolone 1g/d for 3 days  - oral steroids 1mg/kg/d  - oral methotrexate 20mg/week  - Infliximab | NO | OU laser photocoagulation | 8 | OU NLP  OU CRAO, Infliximab 5mg/kg was added, vision stabilized for 6 months | | | OU CRAO | |
|  | 58/F | OD 20/40  OS 20/40 | OS occlusion of the superior temporal branched retinal vein | Type 1 TA  OS BRVO | - oral steroids 20 mg/d  - methotrexate 30mg every 2 weeks | NO | OS laser photocoagulation | 384 | OS 20/40, disc swelling (steroid glaucoma and cataract) | | | OS cataract and glaucoma secondary to the steroid | |
| Sakthiswary et al 2012 | 20/F | OD LP  OS HM | OU NVD, MA, capillary nonperfusion, cotton wool spots | Type 1 TA  OU TR stage IV | - intravenous pulse  methylprednisolone 1 g daily for 3 days  - oral prednisolone 1 mg/kg/d  - oral methotrexate of 15 mg/week  - oral warfarin | NO | OU PRP | 4 | OU vision remained same | | | NONE | |
| Pelegrin et al 2012 | 42/M | OD 20/25  OS 20/200 | OU NVI, MA, AV, venous dilation, capillary nonperfusion; OS NVD, preretinal and vitreous hemorrhage | Type 1 TA  OU TR stage IV | - oral prednisolone 6 mg/d  - oral methotrexate 20mg/week  - aspirin 100mg/d | NO | OU intravitreal bevacizumab; OD PRP, OS vitrectomy and cataract surgery | 20 | OU regressed NVI, vitreous hemorrhages and retinal neovascularization | | | NONE | |
| Wang et al 2012 | 25/F | OD LP  OS HM | OD exudative retinal detachment, retinal neovascularization, OS cataract, AV | Type 1 TA  OD TR stage IV  OS TR stage III | - oral steroids  - oral cyclophosphamide  - aspirin | NO | OD laser photocoagulation, OS cataract surgery | NA | OD HM  OS 20/320  OD regression of neovascularization and retinal detachment | | | NONE | |
|  | 13/F | OD LP  OS 20/20 | OD vitreous hemorrhage, retinal detachment; OS retinal neovascularization | Type 1 TA  OD TR stage IV  OS TR stage III | - oral steroids  - oral cyclophosphamide  - aspirin | NO | OD vitrectomy, OS laser photocoagulation | 96 | OD CF  OS regression of neovascularization | | | NONE | |
| Author, year | **Age/**  **sex** | **Initial visual acuity** | **Ocular examination** | **Diagnosis** | **Medical therapy** | **Collateral vessels** | **Ocular procedure** | **Follow-up (week)** | **Ocular outcome** | | | **Complication** | |
| Demir et al 2010 | 14/F | OD 20/20  OS CF | OS NVI; OU delayed retinal circulation, microhemorrhages, NVD, MA, | Type 1 TA  OU TR stage IV | - intravenous methylprednisolone 1g/d for 3 days  - oral steroids  - oral methotrexate 10 mg/week | NO | OU PRP | 48 | OU cataract development, OS cataract surgery | | | OU cataract, OS phthisis bulbi | |
| Das et al 2010 | 16/F | OD 20/40  OS LP | OS NVI, MA, AV, non-filling of the tertiary branches of the lower temporal branch retinal artery | Type 1 TA  OS BRAO | - oral prednisolone 40mg/d | NO | OS PRP | 8 | OS 20/120, but synechial closure of the angle | | | OS NVG | |
| Kaushik et al 2005 | 40/F | OD 20/20  OS LP | OS delayed retinal circulation, non-filling of tertiary branches of the central retinal artery, choroidal infarct | Type 1 TA  OS BRAO | - oral prednisolone 40mg/d | NO | NONE | 12 | OS optic disc pallor | | NONE | | |
| Park et al 1999 | 25/F | OD 20/20  OS 20/1000 | OS RAPD, delayed circulation, venous dilation, leakage of fluorescein at the optic disc and fovea | Type 2b TA  OS AION | - intravenous methylprednisolone 1000mg daily  - oral prednisolone 60mg/d | NO | NONE | 16 | OS no visual acuity improvement, improved venous dilation, mild disc pallor | | | NONE | |
| Karam et al 1999 | 24/F | OD 20/20  OS 20/20 | OU venous dilation, MA, AV in the mid-peripheral regions | Type 5 TA  OU TR stage III | - oral prednisone 60 mg/d | NO | NONE | NA | Symptoms (visual loss upon standing) improved | | | NONE | |
|  | 21/F | OD NLP  OS 20/20 | OU sluggish blood flow, cotton wool spots, MA | Type 1 TA  OU TR stage II | - oral prednisone  - oral azathioprine | YES | NONE | 4 | Symptoms (visual loss upon standing) improved | | | NONE | |
|  | 8/F | OD CF  OS CF | OD venous dilation, sluggish flow, macular edema; OS optic atrophy | Type 1 TA  OU AION | - oral prednisone | NO | NONE | NA | Symptoms improved | | | NONE | |
| Author, year | **Age/**  **sex** | **Initial visual acuity** | **Ocular examination** | **Diagnosis** | **Medical therapy** | **Collateral vessels** | **Ocular procedure** | **Follow-up (week)** | **Ocular outcome** | | | **Complication** | |
| Schmidt et al 1997 | 46/F | OD NLP  OS NLP | OU swollen disc; OD hemorrhages, OS a patch of ischemic retina | Type 1 TA  OU AION | - intravenous methylprednisolone 500mg q12h for 6 days  - oral prednisone 80mg/d | YES | NONE | 8 | OD minimal LP  OS NLP  OU fundus no improvement | | | NONE | |
| Paterson et al 1957 | 48/F | OD CF  OS NLP | OD NVD, MA, granular blood column, venous dilation; OS dense cataract, complete retinal artery occlusion | Type 1 TA  OD TR stage IV | - anticoagulant therapy  - vasodilator | NO | NONE | 20 | OD completely blind, OD development of subcapsular cataract | | | OD cataract, Died of cerebral ischemic events | |

TA: Takayasu’s arteritis; TR: Takayasu’s retinopathy; MA: microaneurysms; AV: arteriovenous shunts; NVG: neovascular glaucoma; NVD: neovascularization of optic nerve head; NVI: neovascularization of iris; TRD: tractional retinal detachment; PRP: pan retinal photocoagulation; M: male; F: female; OD: right eye; OS: left eye; OU: both eyes; CF: count finger; d: day; HM: hand movement; LP: light perception; NLP: no light perception; IOP: intraocular pressure; ERG: electroretinogram; AION: anterior ischemic optic neuropathy; BRAO: branch retinal artery occlusion; CRAO: central retinal artery occlusion; BRVO: branch retinal vein occlusion; NA: not applicable.
